# Comparative analysis of the *Mercenaria mercenaria* genome provides insights into the diversity of transposable elements and immune molecules in bivalve mollusks

**DOI:** 10.1186/s12864-021-08262-1

**Published:** 2022-03-08

**Authors:** Sarah Farhat, Eric Bonnivard, Emmanuelle Pales Espinosa, Arnaud Tanguy, Isabelle Boutet, Nadège Guiglielmoni, Jean-François Flot, Bassem Allam

**Affiliations:** 1 Marine Animal Disease Laboratory, School of Marine and Atmospheric Sciences, 100 Nicolls Road, Stony Brook University, Stony Brook, NY 11794-5000 USA; 2 Sorbonne Université, CNRS, UMR 7144 AD2M, Station Biologique de Roscoff, Place Georges Teissier, 29688 Roscoff, France; 3 Université libre de Bruxelles (ULB), Evolutionary Biology & Ecology, Avenue F.D. Roosevelt 50, B-1050 Brussels, Belgium; 4 Interuniversity Institute of Bioinformatics in Brussels - (IB)2, B-1050 Brussels, Belgium

**Keywords:** Hard clam, Genome, Gene duplication, Repeats, Steamer elements

## Abstract

**Background:**

The hard clam *Mercenaria mercenaria* is a major marine resource along the Atlantic coasts of North America and has been introduced to other continents for resource restoration or aquaculture activities. Significant mortality events have been reported in the species throughout its native range as a result of diseases (microbial infections, leukemia) and acute environmental stress. In this context, the characterization of the hard clam genome can provide highly needed resources to enable basic (e.g., oncogenesis and cancer transmission, adaptation biology) and applied (clam stock enhancement, genomic selection) sciences.

**Results:**

Using a combination of long and short-read sequencing technologies, a 1.86 Gb chromosome-level assembly of the clam genome was generated. The assembly was scaffolded into 19 chromosomes, with an N50 of 83 Mb. Genome annotation yielded 34,728 predicted protein-coding genes, markedly more than the few other members of the Venerida sequenced so far, with coding regions representing only 2% of the assembly. Indeed, more than half of the genome is composed of repeated elements, including transposable elements. Major chromosome rearrangements were detected between this assembly and another recent assembly derived from a genetically segregated clam stock. Comparative analysis of the clam genome allowed the identification of a marked diversification in immune-related proteins, particularly extensive tandem duplications and expansions in tumor necrosis factors (TNFs) and C1q domain-containing proteins, some of which were previously shown to play a role in clam interactions with infectious microbes. The study also generated a comparative repertoire highlighting the diversity and, in some instances, the specificity of LTR-retrotransposons elements, particularly Steamer elements in bivalves.

**Conclusions:**

The diversity of immune molecules in *M. mercenaria* may allow this species to cope with varying and complex microbial and environmental landscapes. The repertoire of transposable elements identified in this study, particularly Steamer elements, should be a prime target for the investigation of cancer cell development and transmission among bivalve mollusks.

**Supplementary Information:**

The online version contains supplementary material available at 10.1186/s12864-021-08262-1.

## Background

The hard clam, *Mercenaria mercenaria*, also known as the northern quahog, is a member of the Veneridae family (Mollusca, Bivalvia) and is native to the North American Atlantic coast, ranging from Maritime Canada to Florida. It has been introduced to Europe (i.e., United Kingdom, France) and to China for marine resource restoration and aquaculture purposes. *M. mercenaria* has a ubiquitous distribution and is physiologically tolerant to wide ranges of temperature and salinity [1, 2]. The species supports a productive shellfish industry along the east coast of the United States (over 8 million pounds, valued at over 60 million US dollars; NMFS 2018) and represents the most economically important marine species in several states. The aquaculture of the species is also rapidly growing in China [3]. In addition to their economic value, hard clams, like other suspension feeding bivalves, play an important ecological role in benthic-pelagic coupling by transferring energy to the benthos and cycling large amounts of particulate matter [4–7].

Multiple biological and environmental stressors have been associated with large-scale hard clam mortality events, leading to major economic losses. For example, clam populations throughout the Northeastern U.S. have suffered severe mortality events due to a fatal disease caused by a protistan parasite called *Mucochytrium quahogii* (formerly known as QPX, [8]). Previous work showed that clam’s susceptibility toward QPX depends upon the origin of the broodstock, suggesting a genetic basis for clam resistance. Our prior research allowed the identification of transcriptomic signatures [9, 10] and genetic features (single nucleotide polymorphisms, [11]) associated with clam resistance to QPX disease. Similarly, significant mortality events have been reported in clams from other areas along the east coast of the U.S., often associated with stressful environmental conditions, particularly low-salinity events (freshets [2, 12]) and heat waves [13], and survival to these stressors is also thought to be linked to clam’s genetic background.

An emerging disease that has been increasingly affecting *M. mercenaria* populations in the Northeastern U.S. during the last decade is disseminated neoplasia [14]. As in other bivalve species, this disease (which is also called leukemia or sarcoma [15]) is characterized by the presence of large anaplastic cells in blood vessels and sinuses throughout the connective tissues of the affected animals. Bivalve neoplastic cells share several morphological similarities with malignant vertebrate cells, including the presence of a hyperchromatic, hypertrophied nucleus, altered Golgi complexes and swollen mitochondria [16, 17]. An important feature identified in neoplastic cells in bivalve mollusks is an upregulation of transposases and transposable elements expression [18, 19]. Recent investigations in the softshell clam (*Mya arenaria*, another member of the Veneridae) showed that disseminated neoplasia is transmissible, making it one of a few transmissible cancers known in nature [20–22]. These authors further demonstrated that neoplastic cells derived from some bivalve species (e.g., the clam *Venerupis corrugata*) can affect other bivalve species (e.g., *Polititapes aureus,* another sympatric clam species). The same authors identified in *M. arenaria* a novel retrotransposon they called *Steamer* (member of the Ty3/Gypsy superfamily) that displayed high copy numbers and activity in neoplastic cells [23]. Even though it remains unclear whether disseminated neoplasia in *M. mercenaria* is transmissible or not, the similarity in pathogenesis among bivalves and similarity of some morphological and molecular features to those seen in mammals makes bivalves an appealing model to investigate retrotransposon-related oncogenesis in animals.

In this context, the availability of high-quality genomic resources for *M. mercenaria* is not only required to expand the repertoire of genomic resources on understudied bivalve species, but is also expected to have a strong impact on basic (e.g., oncogenesis and cancer transmission, adaptation biology) and applied (clam stock enhancement, genomic selection) research. For these reasons, previous effort has been made to characterize the hard clam genome. For instance, our previous work generated a first draft assembly that was used as a reference to identify genetic features associated with clam resistance to QPX disease [11]. That draft was 2.4 Gb in size, markedly larger than the fluorometric estimate of 1.956 Gb. More recently, Song et al. [24] produced another genome assembly generated from a *M. mercenaria* specimen (designated YKG) derived from a clam stock introduced from the U.S. to China several decades ago. This assembly showed an expansion in the baculovirus inhibitor of apoptosis repeat-containing protein genes, which is an important gene family with roles in apoptosis, cytokine production and chromosome segregation [25], and was hypothesized by the authors to contribute to hard clam resilience to stressors. In the current work, we built on these previous genomic investigations and produced a chromosome-level assembly from a clam derived from the native range of the species using a combination of long and short-read sequencing technologies. We then contrasted our novel assembly with that produced by Song et al. [24] to assess the potential existence of chromosomal rearrangements between genetically segregated clam stocks, a first such comparison in the Venerida. Further, we performed a comparative analysis that encompasses other members of Bivalvia to underline gene family expansion, tandem duplication and Steamer elements diversity associated with the *M. mercenaria* genome, particularly for genes highly suspected to be involved in cancer development and spread in these organisms.

## Results

### Chromosome-level genome assembly

**Fig. 1:**
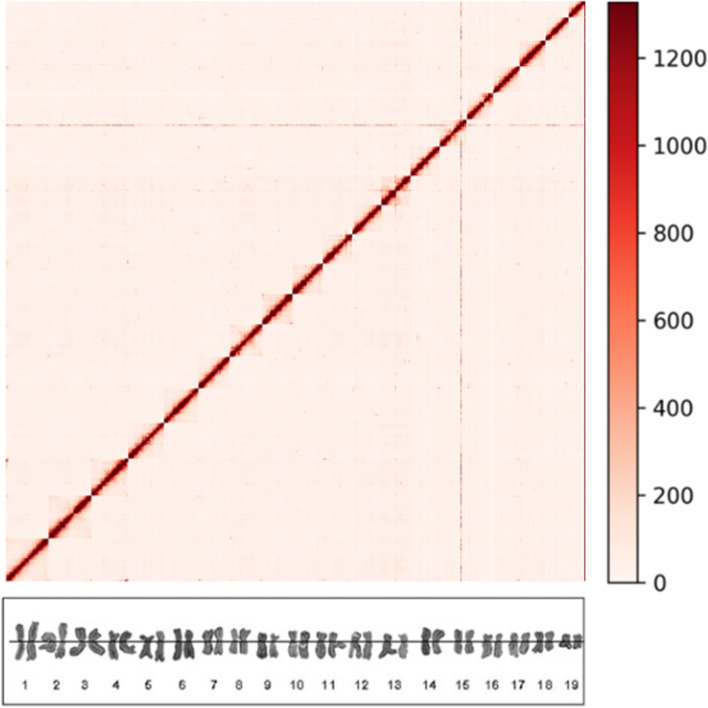
Contact map of the *Mercenaria mercenaria* genome assembly. Map generated from Hi-C data showing sequences interaction points in chromosomes (red dots). The color bar indicates the density of contact. The associated karyotype (from Wang and Guo, 2007 [26]) is shown under the plot

The genome of *M. mercenaria* was sequenced and assembled using a combination of sequencing technologies, resulting in an assembly size of 1.86 Gb with a N50 size of 83 Mb and a GC content of 35%. We reconstructed 19 chromosomes with the help of Hi-C data (Fig. 1) and no contamination was detected in the final DNA sequences (Fig. S1). The completeness of the genome assembly scored 76.4% using the Mollusca database from BUSCO and 91.8% using the Metazoan database, ranking our assembly among the best ever obtained for Venerida (Table S1).

**Fig. 2:**
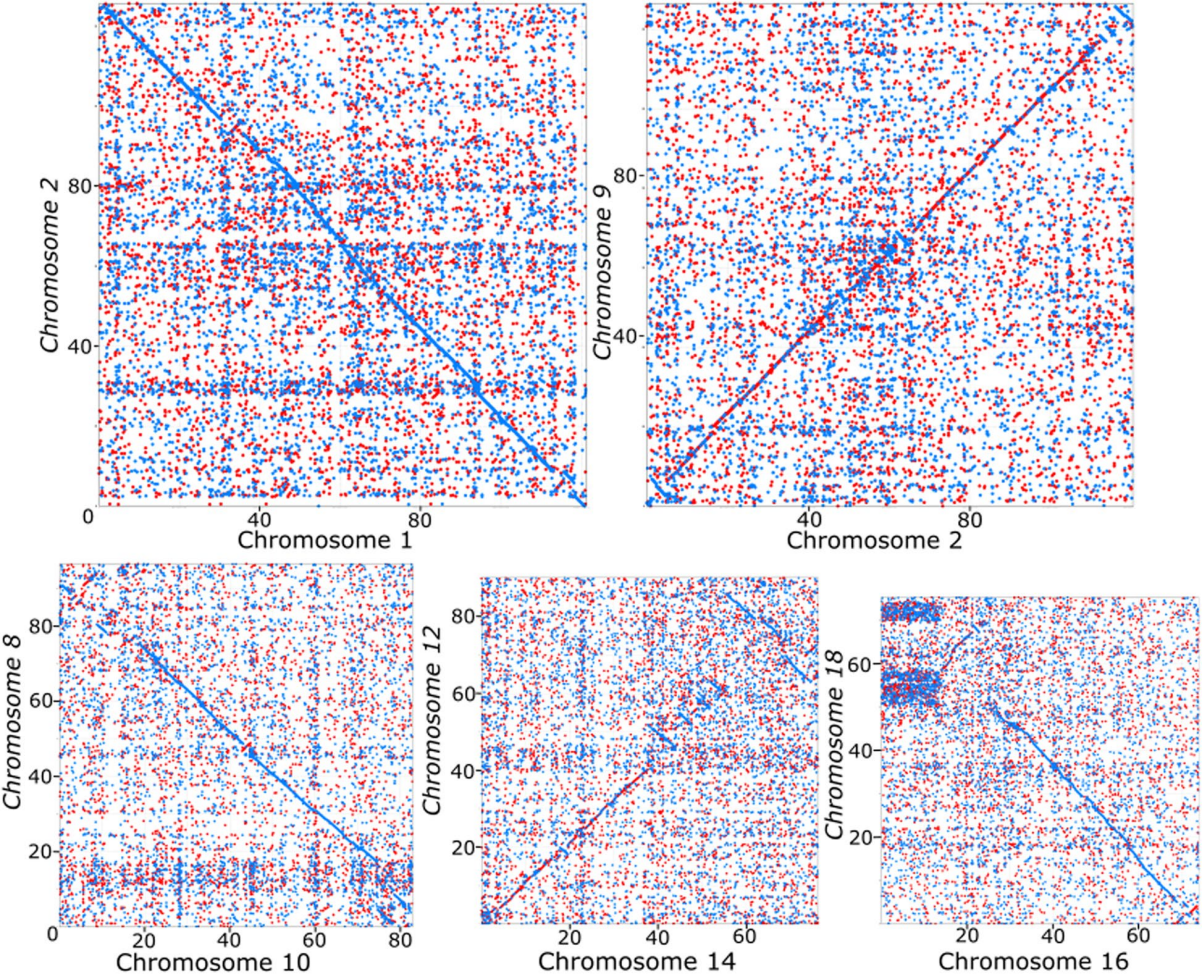
Dotplot contrasting the chromosomes from the two *Mercenaria mercenaria* genome assemblies. All x-axis represents the nucleotides number (in Mb) of *M. mercenaria* chromosomes (generated in this study) and all y-axis represents the nucleotides of YKG chromosomes [24]. A red point represents a forward match and a blue point a reverse match

Comparison of our assembly to another assembly published recently (*M. mercenaria* YKG, [24]) revealed inversions and translocations (Fig. 2). Most of the chromosomes showed minimal differences (Fig. 2, top panels) whereas chromosomes 10, 14 and 16 displayed large structural differences as well as possible duplication events (Fig. 2, bottom panels).

Repeated elements were annotated in both *M. mercenaria* assemblies (*M. mercenaria* and *M. mercenaria* YKG) using the same pipeline method for proper comparison. We detected similar content of various repeated elements (Table 1). Overall, around 45% of both genomes were made up of repeated elements, of which less than 25% of the assemblies were unclassified repeats. The remaining repeats included around 10% of retrotransposons (including half LTR-retrotransposons), 6% of DNA transposons and 4 to 5% of Rolling-circles elements. In addition, 10% of both genomes were found to have satellite DNA elements.

**Table 1:** Repeated sequences in *Mercenaria mercenaria*

	***Mercenaria mercenaria***			***M. mercenaria YKG***		
	Genomic content (%)	Nb of copies	Nb of base pairs	Genomic content (%)	Nb of copies	Nb of base pairs
**Retroelements**	**9.68**	**339,509**	**179,875,339**	**9.74**	**305,795**	**174,196,718**
** SINEs**	1.98	213,809	36,728,732	1.54	179,455	27,466,966
** Penelope**	0.50	11,273	9,261,821	0.36	7596	6,410,976
** LINEs:**	**3.33**	**51,152**	**61,848,151**	**3.20**	**47,180**	**57,211,122**
** L2/CR1/Rex**	1.56	26,146	28,997,539	1.41	22,581	25,216,806
** RTE-X**	0.86	12,800	15,956,581	0.74	11,182	13,148,040
** R1/LOA/Jockey**	0.57	7243	10,588,945	0.59	7013	10,529,781
** L1/CIN4**	0.28	4000	5,126,704	0.32	4497	5,795,447
** R2/R4/NeSL**	0.01	153	168,036	0.01	164	226,620
** RTE/Bov-B**	0.01	119	113,581	0.01	185	172,540
** CRE/SLACS**	0.00	112	80,085	0.00	0	0
** Others**	0.03	579	573,523	0.10	1558	1,708,752
** LTR elements:**	**4.37**	**74,548**	**81,298,456**	**5.01**	**79,160**	**89,518,630**
** Gypsy**	3.87	63,016	71,828,997	4.27	66,510	76,423,586
** BEL/Pao**	0.36	5102	6,644,022	0.55	9351	9,821,340
** Copia**	0.07	1425	1,289,690	0.13	2022	2,285,303
** Retroviral**	0.08	2128	1,535,747	0.06	1277	988,401
** Others**	0.00	0	0	0.05	742	928,874
** YR elements:**	**0.22**	**2877**	**4,028,291**	**0.28**	**2883**	**5,084,062**
** Ngaro**	0.14	1741	2,664,318	0.18	1801	3,287,176
** DIRS**	0.07	1136	1,363,973	0.10	1082	1,796,886
**DNA transposons**	**5.74**	**105,943**	**106,712,868**	**6.28**	**109,894**	**112,397,883**
** Maverick**	1.69	7091	31,368,848	1.93	6453	34,529,082
** TcMar/Pogo**	0.90	22,496	16,783,981	0.77	18,042	13,743,894
** hobo/Ac/Tam**	0.62	13,852	11,531,215	0.71	15,005	12,617,816
** Zator**	0.31	10,651	5,838,880	0.20	7691	3,611,369
** Crypton**	0.28	9809	5,274,930	0.46	13,654	8,303,711
** Academ-1**	0.27	2833	5,097,100	0.31	3377	5,558,034
** EnSpm**	0.13	2744	2,455,561	0.15	4019	2,760,579
** MULE/MuDR/IS905**	0.10	1838	1,822,048	0.04	895	662,200
** Harbinger/Tourist**	0.08	2470	1,526,495	0.11	3294	1,934,619
** PiggyBac**	0.00	0	0	0.01	228	181,113
** Others**	1.35	32,159	25,013,810	1.59	37,236	28,495,466
**Rolling-circles**	4.81	389,027	89,393,728	4.06	374,505	72,579,061
**Unclassified:**	**24.97**	**1,659,652**	**463,988,071**	**24.61**	**1,560,995**	**440,132,557**
**Total interspersed repeats:**	**45.20**		**839,970,006**	**44.69**		**799,306,219**
**Small RNA:**	0.19	16,252	3,490,319	0.37	26,862	6,551,201
**Satellites:**	10.40	645,877	193,200,141	10.68	623,224	190,956,718
**Simple repeats:**	0.95	422,909	17,640,334	0.96	399,918	17,236,842
**Low complexity:**	0.17	65,697	3,083,823	0.16	58,512	2,789,745

Copy number (Nb) and genomic content of the repeated sequences in *M. mercenaria* genomes generated following the annotation pipeline implemented in this study

### Genome annotation and comparison between strains

Genome annotation yielded 34,728 predicted protein-coding genes in *M. mercenaria*, which was only slightly more than the recently published annotation of this species (*M. mercenaria* YKG, 34,283 genes; Song et al., 2021) but higher than other members of the Venerida order (Table S2). The quality metrics of this annotation was higher than the previously published annotation (85% vs 78% of completed BUSCO sequences in our annotation compared to *M. mercenaria* YKG, Table S2). Gene coding regions in *M. mercenaria* were on average smaller than those found in other members of the Veneridae family but were similar to those from other members of the Venerida order (*Archivesica marissinica* and *Lutraria rhynchaena*). Finally, the gene density in our genome assembly (coding sequence coverage of 2.1%) is the lowest across Bivalvia after *Modiolus philippinarum* which, at 2.6 Gb, is the largest genome in Bivalvia described so far.

Using the Best Reciprocal Hit (BRH) method [27], 66 and 67% of the total genes from our clam and *M. mercenaria* YKG, respectively, were identified as orthologs with a median identity percent of 98.9% (Fig. S2); however, this method is known to underestimate the total number of orthologs [27] (Table S3). Using OrthoFinder [28], 84 and 89% of the total genes (the current annotation and *M. mercenaria* YKG annotation, respectively) had orthologs. On the one hand, the number of copies of a gene varied between both annotations, with more duplications found for fewer orthologs in our current assembly in comparison to the YKG assembly (which displayed more orthologs but with fewer duplications for each; Table S3, Fig. S3). On the other hand, more genes from our annotation (5629) were not reported in *M. mercenaria* YKG annotation compared to 3939 genes from *M. mercenaria* YKG that were not found in our assembly. Among these 3939 genes, 1179 genes had an associated GO term, the most abundant of which being related to protein binding (GO:0005515), oxidation reduction (GO:0055114), ATP binding (GO:0005524), integral component of membrane (GO:0016021) and zinc ion binding (GO:0008270). Within the 5629 genes not found in *M. mercenaria* YKG, 1181 had an associated GO term generally representing the same GO terms as listed previously (Table S4).

Regions displaying inversion and translocation events in chromosome 10, 14 and 16 were found to have specific GO term enrichments. For instance, the translocated region in chromosome 10 had the terms “glycerol-3-phosphate catabolic process” (GO:0046168) and “fatty acid biosynthetic process” (GO:0006633) as significantly enriched (*p* < 1.10^− 3^). In chromosome 14, only the GO term “nuclear-transcribed mRNA catabolic process, nonsense-mediated decay” (GO:0000184) was significantly enriched while in chromosome 16, fatty acid beta-oxidation (GO:0006635), oxidation-reduction process (GO:0055114), transposition, DNA-mediated (GO:0006313) and DNA integration (GO:0015074) were enriched.

### Gene duplication and expansion in *Mercenaria mercenaria*

Duplication events were detected in *M. mercenaria* genome (Table S5). Gene duplication analysis combined with a GO term enrichment analysis revealed 11 GO categories including terms related to immunity such as “immune response” (GO:0006955) and “activation of innate immune response” (GO:0002218), molecular signals, cell adhesion and transport such as “G protein-coupled receptor signaling pathway” (GPCR, GO:0007186), “homophilic cell adhesion via plasma membrane adhesion molecules” (GO:0007156), and “transmembrane transport” (GO:0055085, Table S6). The three most significantly enriched GO terms of duplicated genes were “immune response”, “G protein-coupled receptor signaling pathway” and “homophilic cell adhesion via plasma membrane adhesion molecules”. Genes related to immune response (GO:0006955) were annotated as Tumor Necrosis Factor (TNF) for 77 genes and one Tumor Necrosis Factor Receptor (TNFR) using domains annotation method. Among these, 67 TNF were found on Chromosome 7 (10 segmental, 29 tandem, 26 proximal and 2 dispersed) and 2 on Chromosome 14 in tandem duplication; the remainder (8 genes) were in contigs (Table S5). Among the 743 genes related to “G protein-coupled receptor signaling pathway”, 78 were found in segmental duplication, 205 in tandem and 79 dispersed duplications (Table S5). Within the 110 genes related to “homophilic cell adhesion via plasma membrane adhesion molecules”, 6 were segmental, 42 were in tandem and 26 were proximal duplications.

**Fig. 3:**
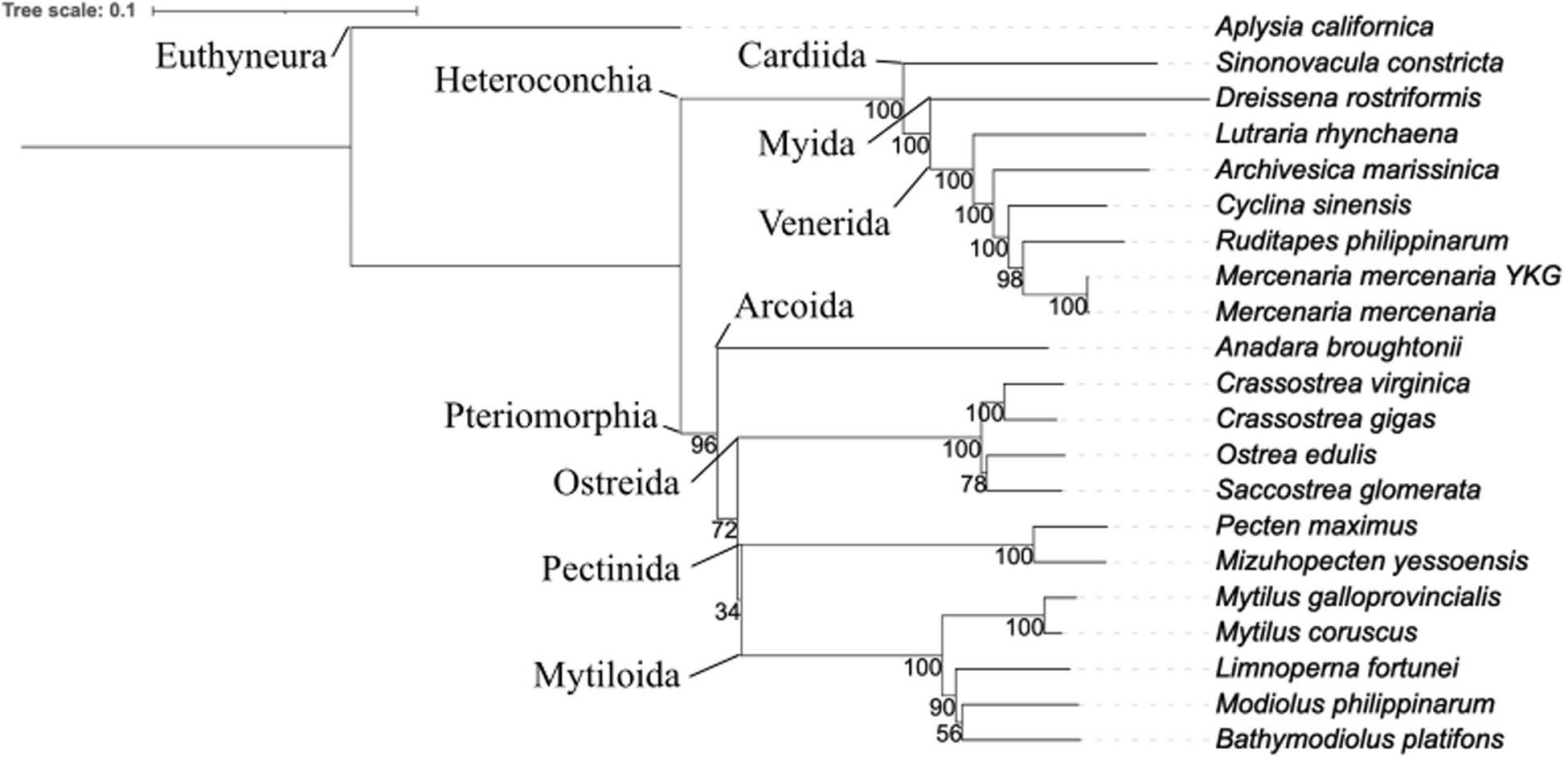
Phylogenetic tree of Mollusca species. Tree of Mollusca species considered in this study based on single copy gene clusters (14 OGs). The tree scale is 0.1 and the bootstrap is represented at each node

To provide a comparative assessment of gene expansion in *M. mercenaria*, we analyzed gene expansions across publicly available assemblies of Bivalvia genomes. For that, OrthoFinder was used on 20 species (including both *M. mercenaria* strains). OrthoFinder clustered 93.8% of the 842,919 genes, which were assigned to 49,574 orthogroups (OGs) where 14,096 were species-specific and 3654 had all species represented. From the 14 OGs having a single copy, we generated a phylogeny (Fig. 3). 3654 OGs had all the considered species represented (Fig. S4). Within the Venerida order, most OGs were found shared between both *M. mercenaria* (2529), then both shared orthogroups first with *Ruditapes philippinarum* (367), then *Cyclina sinensis* (260) and finally shared with the other members of the Venerida order (177) following the phylogeny (Fig. 3). More orthogroups were found to be specific to *M. mercenaria* (2529) than all other Venerida (the second being *A. marissinica* with 339 OGs), highlighting more diversification in species-specific gene families in the hard clam.

Expanded genes were detected in *M. mercenaria* (Table S7) and categorized in 3 groups. We counted 90 orthogroups where the expanded genes in *M. mercenaria* were specific to this species (no orthologs detected in any other considered species). Most of these OGs contained genes related to binding proteins. The most expanded orthogroup was the receptor protein GPCR (27 genes), followed by genes containing a Sushi/SCR/CCP domain (14 genes). A gene family of protein-tyrosine phosphatase-like enzymes, which can create novel recognition motifs for protein interactions and cellular localization, affect protein stability, and regulate enzyme activity, was also expanded (13 genes). Other expanded OGs included those related to immunity, such as Heat Shock protein 70 family, Inhibitor of apoptosis family and proteins containing Toll/interleukin-1 receptor (TIR) domain. Additionally, 26 OGs specifically expanded in *M. mercenaria* remained uncharacterized.

The second expanded group represented gene families having more copies in *M. mercenaria* as compared to the average number of copies in all other species. The most expanded gene in this group was predicted to encode a Ficolin-2-like protein, which displays a carbohydrate binding domain and opsonic activities and is a primary player in the activation of the lectin complement pathway of innate immunity. The number of copies of this family in *M. mercenaria* is similar to that found in *Crassostrea gigas* and *Ostrea edulis* as well as in both *Mytilus* species. The second most expanded gene in this group was also a lectin (C-type lectin) that displayed more copies in *M. mercenaria* as compared to all other species excluding *M. philippinarum* and *Mytilus galloprovincialis*.

The third group of expanded genes represented those displaying a higher number of copies in *M. mercenaria* as compared to the mean number of copies from all other Venerida species. In this group, we found genes related to heat shock protein, transporters (Sodium neurotransmitter symporter), receptors (Toll-like, GPCR) but also immune related molecules such as Toll-like receptor 4 and C1q domain containing proteins.

### Tumor necrosis factor and C1q

Genes belonging to TNF and C1q were manually curated in order to better analyze domain content and evolution because of the role of these genes in immunity and cancer regulation. TNF genes were considered if they had a TNF domain (IPR006052) or were within an orthogroup having genes containing a TNF domain (Table S8), resulting in a total of 76 genes in *M. mercenaria*. As described above, these genes were mostly duplicated in tandem on the genome although some were also found in segmental duplication (Table S8). Ortholog analysis clustered TNF members in 13 distinct OGs (Table S8). One OG (OG0000926) had all species represented where *M. mercenaria* was not the species having most represented copies (Table S9). Four OGs had representative members from the Bivalvia with the exception of the Pectinida family, including 2 OGs (OG0000639 and OG0000960) showing markedly higher numbers of representatives in *M. mercenaria* (at least 2 times more copies except *L. rhynchaena*) as compared to other species. Four other OGs were specific to the Heteroconchia including 3 members specifics to the Venerida. Lastly, 4 OGs were found to be specific to *M. mercenaria* with a total of 5 genes found in our assembly. Most duplicated genes in *M. mercenaria* compared to all other species were found in two OGs specific to Bivalvia and one specific to *M. mercenaria* (i.e., no ortholog genes found in any other species).

**Fig. 4:**
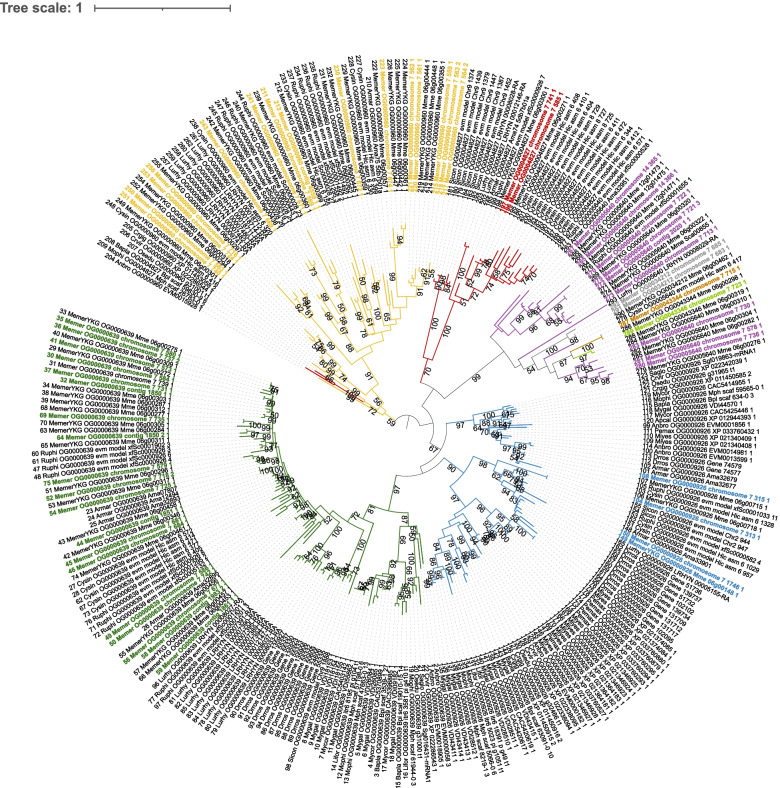
Phylogenetic tree of TNF domains. TNF domains from all identified genes were extracted and used to generate a multiple alignment and a tree using MAFFT. The tree was drawn using iTOL. Colored branches represent the different orthogroups (the colors correspond to those of Table S8. Label colors also represent OG but highlighting only genes belonging to our *Mercenaria mercenaria* assembly

Once all the genes had been manually curated, we identified the domains on each gene and counted the number of transmembrane (TM) domains (Table S8). All but one gene (75 out of 76) had exactly one TNF domain. While we found one TM domain in most (56) of the genes, we identified 2 TM domains in one gene, and none in the remaining (19) genes. The number of TM domains was not related to the orthogroup clustering. Subsequently, TNF domains were extracted to perform a phylogenetic analysis (Fig. 4) that allowed the clustering of the domains into 6 families supported by bootstrap values higher than 70%. Most of these genes were clustered similarly to the OGs excluding OG0000926, which segregated into one branch having only members belonging to *M. mercenaria* set apart from two other branches. The orthogroups OG00005640, OG0034212, OG0043344 and OG0043346 formed a monophyletic group with a bootstrap of 100%, with the first OG specific to Venerida and last three specific to *M. mercenaria* (Fig. 4).

**Fig. 5:**
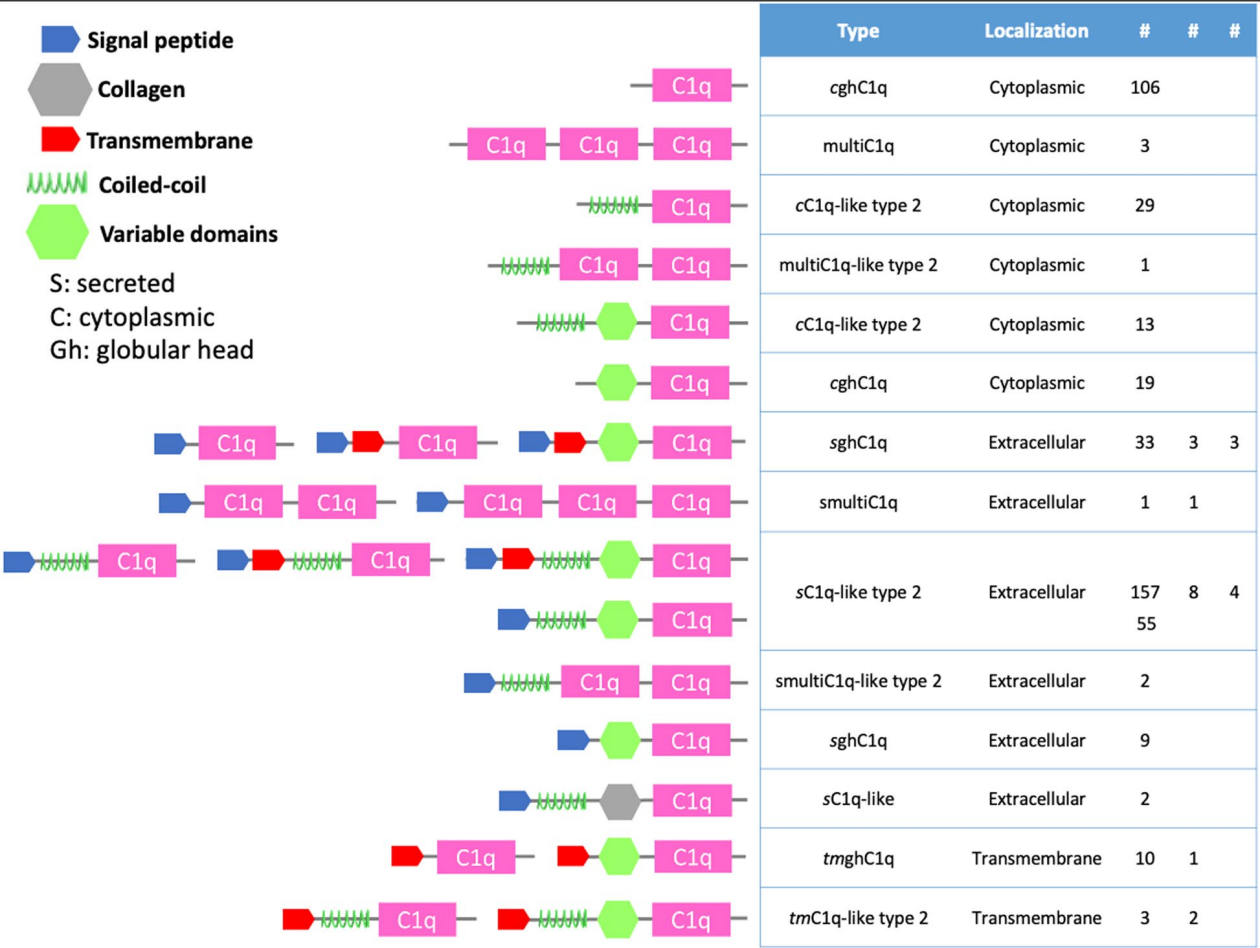
C1qDC proteins in *Mercenaria mercenaria*. Schematic structural domain representation of putative C1qDC proteins from *M. mercenaria*

Similarly to TNF genes, C1qDC genes were considered and manually curated (total of 408 genes in *M. mercenaria*, Fig. S5) if they contained at least one C1q domain (IPR001073) or a tumor necrosis factor-like domain superfamily (IPR008983). The C1qDC genes were defined based on the predicted cellular localization (i.e., cytoplasmic, extracellular, transmembrane) and on the domain organization of the predicted encoded proteins (i.e., presence of signal peptide, transmembrane domain, coil domain, collagen and other domains) following previous recommendations [29, 30] (Fig. 5). A high variability was noted in the structure of the C1qDC genes. The overall C1qDC genes contained a total of 420 domains. A large proportion of these (40% of the total) was classified as secreted sC1q-like type 2 proteins with members characterized by the presence of a signal peptide, a coil domain, sometimes an additional domain and a C1q domain at the N terminal end of the sequence. Another large group of proteins (25%) was described as cytoplasmic globular head (cghC1q) proteins, and only harbors a C1q domain.

The distribution of the genes on the 19 chromosomes was uneven and varied from 3 (chromosome 8) to 47 (chromosome 5). It is noteworthy that 10 C1qDC genes were not found on the assembled chromosomes but on the remaining unassembled contigs. All but one C1qDC genes were duplicated, with most members being tandemly duplicated (46.1%), followed by dispersed duplication (31.1%) (Fig. S5). Segmental duplication (affecting 10.5% of the overall number of C1qDC genes) was also observed on chromosomes 19 (52% of the duplicated genes identified on that chromosome), 16 (30.6%) and 18 (25%).

The C1qDC genes detected in *M. mercenaria* were compared to those identified among the 20 other molluscan genomes investigated, including the *M. mercenaria* YKG genome. Ortholog analysis produced 136 OGs. More than 50% of the *M. mercenaria* C1qDC genes were present in 18 OGs (Fig. S6) and 74 OGs contained sequences unique to *M. mercenaria* (e.g., OG0016876).

To simplify the amino acid pattern characterization, consensus sequences were deduced (majority amino acid rule) from genes belonging to *M. mercenaria* in each OGs and aligned to reveal pattern conservation (Fig. S7). Results showed that the C1q domain was made up of about 130 amino acids and contained residues that are highly conserved (i.e., G44, G52, Y54, F143) as well as moderately conserved ones (i.e., A7-F8, F28, N34, G36, Y39, F46, P49, F56, G145, L146).

### Steamer elements

Given the apparent sensitivity of *M. mercenaria* to hemic neoplasia (leukemia [14]), we focused our investigations on Steamer LTR-retrotransposon family as it was associated to neoplasia in other bivalve species [31]. In order to identify Steamer elements in mollusk genomes, LTRharvest was used and its output was integrated in phylogenetic analyses (Fig. S8). For this purpose, two types of sequences were used: (i) either a consensus devoid of possible insertions in the case of a cluster of elements previously defined by Uclust, (ii) or isolated (single) sequences that did not cluster with any other elements. Predicted Reverse Transcriptase/Ribonuclease-H (RT/RNaseH) domains were translated and a BLAST approach was used to only retain LTR-retrotranspons potentially belonging to the C-clade. To assess the diversity of C-clade elements, a phylogenetic tree was built based on 737 sequences from bivalves (in addition to 50 reference elements essentially from mollusks, annelids and echinoderms). Almost all sequences are included in 21 differentiable branches, including the SURL elements branch as well as a branch of 21 sequences containing the reference *Steamer* elements of *M. arenaria* and *Ensis directus* [23]. Branches were defined on criteria similar to those used in our previous analysis to define clades [32]: (i) to be shared by several species and (ii) to have a monophyletic group with a bootstrap value greater than 80. The number of identified sequences varied between different branches, with the branch 4 m (for mollusks, as opposed to the 4a of annelids) being the largest with 176 sequences, followed by C5 and C7 (61 and 56 sequences, respectively). The branch containing the Steamer elements (bootstrap support of 100%) has (19 sequences), about twice that of the SURL elements (8 sequences). It appears that for almost all branches the number of sequences coming from clusters is similar to the number of single sequences.

**Fig. 6:**
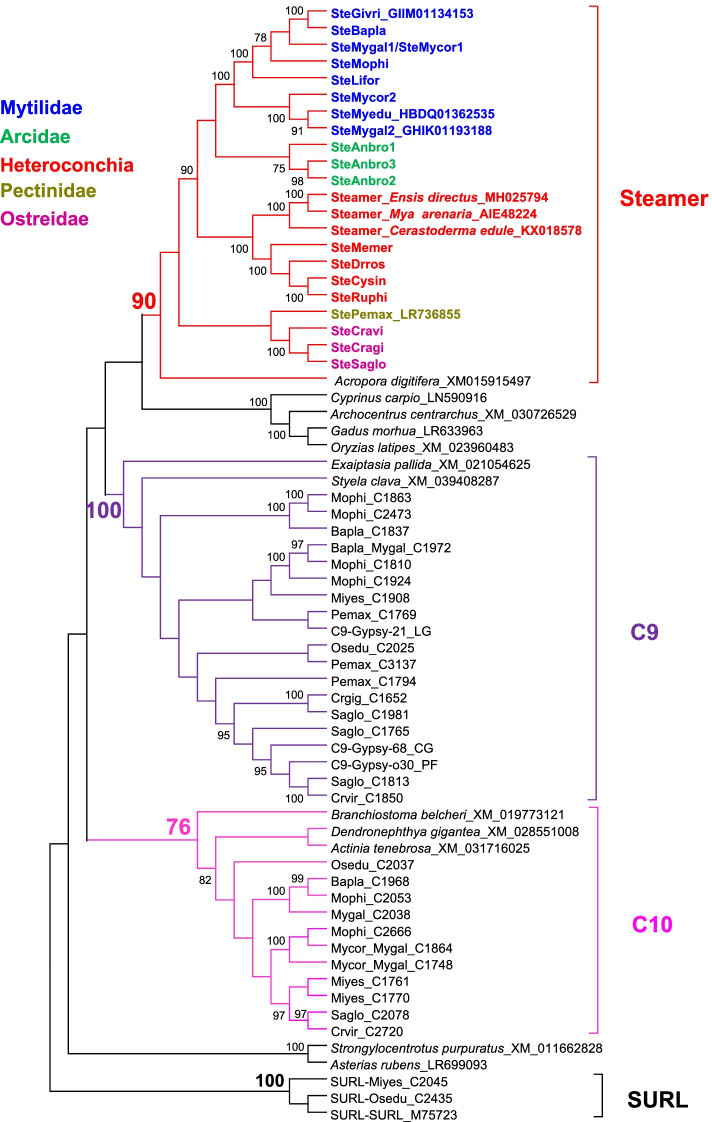
Phylogenetic relationships of Steamer retrotransposons and their close groups. This tree is based on Neighbor-Joining analysis [33] of RT/RNaseH domain amino acid sequences. Node statistical support values come from non-parametric bootstrapping using 100 replicates and only those > 75% are shown. The three C-clade branches are indicated in color. Sequences from *Lottia gigantea* (LG), *Pinctada fucata* (PF) and *Crassostrea gigas* (CG) are included as known references of C9 groups [32], and SURL elements included as outgroup. Sequences obtained from TBLASTN searches are labelled by their accession number. For Steamer elements members of different taxa are color coded as shown. Givri: *Gigantidas vrijenhoeki*; Bapla: *Bathymodiolus platifrons*; Mygal: *Mytilus galloprovincialis*; Mycor: *Mytilus coruscus*; Mophi: *Modiolus philippinarum*; Lifor: *Limnoperna fortunei*; Myedu: *Mytilus edulis*; Anbro: *Anadara broughtonii*; Memer: *Mercenaria mercenaria*; Drros: *Dreissena rostriformis*; Cysin: *Cyclina sinensis*; Ruphi: *Ruditapes philippinarum*; Pemax: *Pecten maximus*; Cravi: *Crassostrea virginica*; Cragi: *Crassostrea gigas*; Saglo: *Saccostrea glomerata*

The Steamer family branch was part of a polytomy with two other closely related branches, which we called C9 and C10 (Fig. S8). To provide a more detailed characterization of this group, we reconstructed a second phylogenetic tree focused on these three branches for which the sequences were cured and confirmed as belonging to different elements (Fig. 6). This yielded 14 Steamer sub-families (set of copies of the same element within a species) detected by LTRharvest in 12 bivalve genomes. In addition, there were one sub-family generated from *Crassostrea virginica* and one from *R. philippinarum* in which deleted Steamer elements were recovered during RepeatMasker searches on the nine genomes devoid of complete Steamer elements. Finally, no Steamer element could be detected in one third of the bivalves analyzed. We also looked for potential Steamer elements more broadly and systematically by searching the NCBI nucleotide database with the same conserved RT/RnaseH domain from the *Steamer* element of *M. arenaria*. Fifteen sequences were so selected from organisms belonging to different phyla.

The Steamer family was well supported (bootstrap 90%) and included 23 elements derived from bivalves. These elements were named with the prefix “Ste” for “Steamer”, followed by a code referring to the name of the species and a number in case of more than 1 sub-family. The analysis of the genomes revealed only one Steamer sub-family per species except for *Mytilus coruscus* and *Anadara broughtonii* with 2 and 3 sub-families, respectively, even though BLAST searches on public databases also revealed a second sub-family in *M. galloprovincialis*. Sub-families are clearly grouped according to host classification and we found 5 groups of bivalves with the Mytilidae, an Arcidae, the Heteroconchia, a Pectinidae, and the Ostreidae. Among the sequences recovered from NCBI, a part was found in the other two branches C9 and C10 (still well supported with bootstrap of 100 and 76, respectively), with the fish elements forming an independent monophyletic group, as do the elements of a sea urchin and a starfish. Only one element of the coral *Acropora digitifera* appeared to belong to the Steamer family, even if it was clearly separated from the other elements. It thus seemed that the elements of the Steamer branch were found almost exclusively in bivalves.

Once the Steamer elements were well defined according to the phylogenetic analysis, we specified their characteristics by comparing the sequences of the 16 new bivalve sub-families to that of the *Steamer* of *M. arenaria* (Table S10). For each sub-family, there was only a small number of “full-size” copies having their two LTRs (9 at most), so the characteristics were established either on all the available sequences or on a consensus sequence (suppression of indels). The copies detected by LTRHarvest showed quite a large variation in size within the same sub-family due to indels; but all were smaller than the reference *Steamer* (4968 bp). Consensus elements ranged in size from 4899 bp for SteSaglo to 4543 bp for SteBapla (whose copies were quite corrupted). The elements are flanked by LTRs from 153 bp (SteAnbro3) to 219 bp (SteSaglo). The size of the LTRs varied a little between sub-families but also between copies for an average of 187 bp close to the size of the reference *Steamer* element from *M. arenaria* (177 bp). These LTRs all started with the TGTAACA motif but ended with a more variable motif whose majority consensus (TTAAACA) was very close to that of the reference *Steamer* (ATAAACA). The 5′ LTR was immediately followed by the 12 bp Primer Binding Site previously described for *Steamer* as complementary to the 3′ end of the Leu tRNA of the purple sea urchin (TGGTGTCAGAAG), even though one or two substitutions were noted on the last three bases. At last, various PolyPurine Tract sequences were well recognizable upstream of the 3′ LTR. The structure of all new Steamer elements was thus very similar to that of the *M. arenaria* element, with which they also shared the diverse gag-pol motifs [23]. Indeed, the coding sequences were very similar, with a minimum of 55% amino acid identity between elements on the whole RT/RnaseH domain set (70% on average). The coding *Gag* and *pol* regions were grouped into a single ORF that was still intact for only 7 sub-families, including SteMemer. Five other sub-families had copies with only 1 or 2 frameshifts, while the ORF in the remaining subfamilies appears highly corrupted or deleted.

Once the Steamer elements were clearly identified, their copy number and genomic proportion were estimated in the 13 species where they were present (Table 2). Only 3 species had more than 5 full size copies, with 7, 11 and 15 detected by LTRHarvest in *M. mercenaria*, *M. coruscus* and *A. broughtonii*, respectively. These values hardly changed when looking at copies larger than 4 kb found by RepeatMasker (63 copies out of all genomes, 52 of which were already recognized by LTRHarvest); this underlined that the majority of the large copies still have their two recognizable LTRs. The number of deleted copies increases only slightly when the size threshold is brought down to 2 kb. Finally, the total number of loci with a potential Steamer insertion remained low with less than a hundred sites in six genomes (including *R. philippinarum* where SteRuphi is almost absent), 156 in *M. mercenaria*, and a maximum of 545 sites for *A. broughtonii*. In the latter, copies were evenly distributed among the three SteAnbro sub-families, as opposed to *M. coruscus* where the SteMycor2 sub-family dominated. These low copy numbers represented a very small proportion of the genome size, often close to 0.01% except for *Saccostrea glomerata* and *A. broughtonii* with 0.03 and 0.04%, respectively. The Steamer elements of *M. mercenaria* are thus among the closest to those of *M. arenaria* in terms of phylogeny and characteristics; and the SteMemer remains one of the best represented subfamilies with potentially active copies having an identifiable single ORF.

**Table 2:** Steamer elements subfamilies found across Bivalvia

Host species	Family	Number of copies				Genomic proportion (%)	Base pair masked in genome	Average length
		LTRH	RM > 4 kb	RM > 2 kb *	RM			
*Mercenaria mercenaria*	SteMemer	7	7	8	156	0.01	148,438	951
1858 Mb								
*Mercenaria mercenaria* YKG	SteMemer	5	6	7	166	0.01	148,344	894
1788 Mb								
*Ruditapes philippinarum*	SteRuphi	0	0	3	22	0.00	21,751	989
1123 Mb								
*Cyclina sinensis*	SteCysin	2	2	1	64	0.01	54,243	848
903 Mb								
*Dreissena rostriformis*	SteDrros	5	5	5	65	0.01	78,925	1214
1242 Mb								
*Anadara broughtonii*	SteAnbro1	5	5	0	176	0.01	104,324	593
885 Mb	SteAnbro2	5	5	5	153	0.01	114,234	747
	SteAnbro3	5	7	15	216	0.02	177,833	823
*Crassostrea gigas*	SteCragi	1	1	10	99	0.01	96,188	971
648 Mb								
*Crassostrea virginica*	SteCavi	0	0	1	70	0.01	42,104	601
685 Mb								
*Saccostrea glomerata*	SteSaglo	4	5	9	284	0.03	203,715	717
788 Mb								
*Bathymodiolus platifrons*	SteBapla	3	3	8	216	0.01	168,122	778
1658 Mb								
*Modiolus philippinarum*	SteMophi	2	2	13	244	0.01	195,023	799
2630 Mb								
*Limnoperna fortunei*	SteLifor	1	5	19	142	0.01	161,653	1138
1673 Mb								
*Mytilus coruscus*	SteMycor1	2	4	3	96	0.00	93,720	830
1904 Mb	SteMycor2	9	11	6	265	0.01	256,509	968
*Mytilus galloprovincialis*	SteMygal1	1	1	10	81	0.01	92,780	1145
1282 Mb								

Number of copies and genomic proportions of Steamer elements subfamilies were estimated in bivalve genomes using LTRHarvest (LTRH) or RepeatMasker (RM). Copy numbers are given according to the estimation procedure. The star indicates between 2 and 4 kb

## Discussion

### Features of the hard clam genome

The Bivalvia class includes about 9200 extant species, many of which support major fisheries and aquaculture industries. Among these, only 33 species have been sequenced (https://www.ncbi.nlm.nih.gov/), with most of the recent sequencing effort focusing on the production of chromosome-level assemblies. In the Venerida order, 6 species have their genomes assembled [24, 34–37] including one that is not scaffolded at a chromosomal level (*Corbicula fluminea*, [38]). In this framework, understanding the genetic components and features of these organisms can unlock multiple scientific fields, especially for enabling approaches and strategies to understand, prevent and mitigate diseases or to develop aquaculture stocks that resist infections and environmental stressors.

Supported by previous karyotype analyses, we successfully assembled the 19 chromosomes of the hard clam (Fig. 1, [26, 39]). This number is similar to that identified in all chromosome-level assemblies performed in the Venerida order so far [24, 34–37] (Table S1). Our *M. mercenaria* genome assembly has a size of 1.86 Gb (N50 of 83 Mb) and is very close to the genome size predicted previously using flow cytometry (1.96 Gb, [40]). While another chromosome-level genome assembly has been recently produced for *M. mercenaria* by another team [24], our assembly was closer to the predicted genome size and had slightly better BUSCO completeness scores (Table S1). Thus, the hard clam genome produced here represents the third largest genome sequenced to date in the Bivalvia class after the Mytilida order (*M. philippinarum* 2.6Gb [37] and *M. coruscus* 1.9Gb, [41], Table S1). More than half of the genome is composed of repeated elements, including transposable elements that are known to play important roles in genetic changes [42]. This particular feature results in a less gene-dense genome than those of other members of the Venerida, while it has more gene counts but with smaller mean gene length. Our study also highlights the expansion of some gene families, particularly receptor and binding domain-containing genes involved in immune recognition and activation. The expansion of immune-related genes may, at least in part, explain why the average gene size in *M. mercenaria* is small as compared to other bivalves, as previous studies showed that genes related to immune activity are generally small to allow for fast expression since defense responses and receptor agonist activities need to be rapidly expressed to cope with immune challenges throughout the life of an organism [43].

The two hard clam genome assemblies compared at a genomic level in this study derive from two genetically distinct populations. For instance, the clam analyzed in this work came from a well-established clam population in New York while the recently-published YKG strain derives from a small group of genitors introduced from the east cost of the USA to China in the 1990s (precise source unknown). While most of the genomic features identified in both assemblies are similar (total number of genes, average gene length, total number of repeated elements, GC percent, etc.…), gene comparison showed important differences. For instance, 16% of genes predicted in our assembly were not detected in YKG, while 11% of genes identified in the latter were not detected in our assembly. Some of these differences may be explained by technical discrepancies (e.g., assembly parameters, unassembled reads and contig filtering, etc.), while others may derive from authentic biological processes (such as changes induced by invasive elements, or chromosome rearrangement). Massive gene presence-absence variations have been recently described in the Mediterranean mussel *M. galloprovincialis* [44] and similar processes may lie at the heart of differences detected here between *M. mercenaria* and *M. mercenaria* YKG. To our knowledge, this is the first study that uses whole-genome information to compares chromosomal structures in the Venerida and results allowed the identification of significant chromosomal rearrangements in 3 chromosomes. Previous work to characterize chromosomal rearrangements in bivalves used karyotyping and fluorescent in situ hybridization of specific molecular targets and showed high inter- and intra-specific variations in chromosome structures. For example, Thiriot-Quiévreux and Insua (1992) [45] investigated variations in the nucleolar organizer region (NOR) in three oyster species and showed marked differences within and between species in terms of number of NORs per genome, their chromosomal location and their position within karyotypes. Similarly, Insua and Mendez (1998) [46] demonstrated differences in the number of rDNA loci between individuals of the same species (*M. galloprovincialis*), as well as in the location of the rDNA locus between different cells from the same individual. The biological significance of these variations is unclear although previous work in bivalves linked these rearrangements to processes pertaining to local adaptation and possible mechanisms of speciation [47, 48]. In fact, the role of chromosomal inversions in adaptation and speciation has been demonstrated in a broad range of organisms [49] and was shown to contribute to the regulation of gene expression [50] and to new gene formation [51]. Similarly, chromosomal rearrangements are a landmark feature during carcinogenesis [52]. Genomic regions impacted by chromosomal rearrangements in our study were particularly enriched in genes related to catabolism, oxidation processes and DNA transposition and integration. The biological significance of these rearrangements in the hard clam requires further investigations.

Song et al. [24] showed an expansion of apoptosis-related gene families (genes containing Baculoviral Inhibitor apoptosis proteins Repeat) in *M. mercenaria*. Our analysis confirmed these findings, but also showed significant duplications in multiple genes involved in immune responses. These included proteins having recognition, binding and signaling domains suggesting a high diversity of signaling pathways that may help clams adapt to complex environmental landscapes. Duplication and expansion of genes related to immune responses and environmental resilience have been previously described in other Bivalvia species. For example, the pearl oyster *Pinctada fucata* displays a remarkable expansion in heat shock protein 70 and this feature is thought to allow oysters to resist environmental changes. Likewise, our investigations in *M. mercenaria* allowed the identification of two major gene families that were broadly duplicated: tumor necrosis factor (TNF) and C1q domain-containing proteins.

### Tandem duplication and expansion of TNF and C1q domains-containing genes

TNF families and C1q proteins both contain tumor necrosis factor (TNF-like) domains, which are known to play a central role in cell signaling and to trigger the intracellular apoptotic cascade. Members of the TNF family are pro-inflammatory cytokines that were first found to regress tumors in mammalian cells [53]. Because of its important role in immunity, this signaling pathway is now very well described in mammals where it includes 19 ligands and 29 receptors [54]. The TNF cascade is relatively well conserved and various members have been identified in Protostomia [55] including Mollusca where it was shown to be expanded with up to 23 TNF family members in the Pacific oyster *C. gigas* [56]. Here, we also found TNF family members to be expanded in all well assembled Bivalvia genomes considered. In *M. mercenaria*, we found 65 TNF members to be localized in one chromosome (chromosome 17) with nearly half of these being tandemly duplicated. Tandem duplication of TNF was also detected in *C. gigas* [56] but to a much lesser extent than in *M. mercenaria*. As genes being tandemly duplicated were part of the same cluster, it shows a recent duplication possibly suggesting an increased need of this molecule for this species. Overall, clustering of TNF proteins from all 20 species showed a diversity of TNF members in Bivalvia. Two OGs specific to *M. mercenaria* had a common ancestor with an OG specific to Venerida, while two others were divergent from all other OGs. This may suggest a parallel evolution of some TNF members in the hard clam. *M. mercenaria* genome still contains genes having a common ancestor with Bivalvia species, but many of these appear to be significantly more expanded in the hard clam as compared to all other species. The biological significance of TNF diversity in the hard clam is unclear but may support a high ability of this species to fine-tune host response to a broad range of microbial and environmental stressors. It should be noted, however, that our comparison is based on a relatively small number of genomes for which high quality assemblies are available, and drawing robust conclusions will require larger samples.

The C1q domain containing (C1qDC) proteins generally refer to a family of proteins containing a globular head C1q domain (gC1q) that enables the recognition of a broad range of ligands and trigger the activation of the classical complement pathway [57]. The C1qDC proteins have been found in vertebrates [58] where the predominant organization includes a signal peptide followed by a collagen region and a C-terminal C1q domain. These proteins have also been found in the genome and transcriptome of numerous mollusk species, including in bivalves [30, 59–61] where they are much more diversified than in gastropods or cephalopods [60]. Their structure is similar to those encountered in vertebrates [62] except that they very often lack a collagen domain. In bivalves, the C1qDC proteins have been found to be involved in several biological functions, particularly in innate immunity where they mediate pathogen recognition, binding and opsonization [63, 64]. The C1qDC proteins are extremely abundant in *M. mercenaria* (408 curated genes), in line with findings in other bivalve species (e.g. 1589 C1qDC in *R. phillipinarum*, [65]; 476 C1qDC in *C. virginica*, [60]). The most abundant type of C1qDC found in *M. mercenaria* genome is the sC1q-like type 2 type, which is also in line with results found in other species, including *C. virginica* [60]. The considerable expansion of the C1qDC proteins is explained by gene duplication (mostly tandem duplication and to a lesser extent dispersed duplication). This phenomenon, coupled with the retention of a large number of these genes, has been observed in other bivalve species [59, 60, 65]. This increase in the diversity of recognition molecules represents a good solution to the lack of antibody-mediated immunity, especially in bivalves where recognition and binding of non-self entities is important not just for immunity and defense, but also for suspension-feeding [66, 67]. Therefore, a diverse repertoire of recognition molecules can enable the processing of a broad range of microbes, including pathogens alike and food particles. An example in the case of *M. mercenaria* is the mRNA.chromosome_3.664.1 gene, which was found here to have a dispersed duplication: this gene was previously reported to recognize and bind *M. quahogii* [68], an eukaryotic microbe (QPX) that infects *M. mercenaria.*

In many instances gene duplication in eukaryotes implies the activity of transposable elements, including DNA transposons and retrotransposons [69–71]. The next section describes our findings on transposable elements in the *M. mercenaria* genome.

### Repeated elements and steamer family

The classification of transposable elements (TEs) is fairly clear and unambiguous when one looks at the major subdivisions [72]. Thus, on the basis of mode of transposition, structure and sequence similarity, it is relatively straightforward to distinguish the three superfamilies Copia, Bel/Pao, and Gypsy. On the other hand, the classification becomes much more challenging when one is interested in higher resolution classifications such as clades or families. A TE clade refers to a monophyletic group of elements present in different host species, so this term is flexible, deliberately imprecise, and can be used at any level of the classification. Therefore, the definition of a clade is sometimes partly dependent on the author’s decision. But more importantly, the study of new host phyla can greatly enhance or enable the characterization of particular clades by modifying the topology of the phylogenetic tree. For example, in the case of the BEL/Pao retrotransposons, the original Pao clade has been subsequently split into two distinct clades, Pao and Dan [73]. The same problem of ambiguous boundaries can be found when the aim is to establish a classification of families or sub-families. They are sometimes defined on the basis of simple rules of identity of sequences by fixing a threshold beyond which different elements are considered as belonging to the same set (e.g. the 80–80-80 rule, [74]). However, this step can be hampered when the number of elements increases, with sequences that are poorly positioned, as some inactive copies may have strongly diverged from the canonical elements. Therefore, such an approach is sometimes difficult to carry out, so we have preferred to define our elements of the Steamer family on the basis of a phylogenetic analysis.

The reference *Steamer* element [23] is clearly a Gypsy LTR-retrotransposon and its close similarity with the SURL family suggested that it belonged to the C-clade, the largest and most abundant of the twenty or so Gypsy clades described in metazoans [32, 75]. The analysis within this clade revealed 21 clearly individualized groups that we called ‘branches’. Each of these branches could correspond to a family of elements; but this would require confirmation since some of them may still contain several distinct families. On the other hand, in some cases two branches could be considered as part of the same family. Host phylogeny greatly influences the subdivisions as evidenced by the C4m and C4a branches, consisting solely of mollusk or annelid elements, respectively. These two branches could eventually be joined at the common node if we had considered a 70% boostrap threshold (instead of 80%). Things are different for the Steamer, C9 and C10 branches. Even if these branches are closely related, the set is never supported (bootstrap of 52 when considering the whole clade and 54 on the more focused tree); and each includes elements from different mollusks as well as organisms other than bivalves, relativizing the possible influence of phylogeny. This is why we decided to associate the one branch containing the three reference *Steamer* elements with the family of the same name.

We have also deliberately chosen not to use the label “-like element” classically used to characterize TEs close to a known element, but which implies an approximation that does not seem justified here. This could possibly apply to the element of the cnidarian *A. digitifera* but the topology clearly includes it in the Steamer family. Metzger et al. [31] conducted a very extensive search for Steamer-like elements (SLEs) both in Mollusca and more broadly in other organisms. We therefore wanted to know how well some of these SLEs fit our characterization of the Steamer family by looking at where they place in our phylogenetic tree. Concerning the whole set of elements, it is clear that the Metzger et al. [31] work consciously uses ‘SLE’ in a very broad sense since their phylogeny includes SURL elements and is rooted with A-clade elements. Several of our elements obtained by BLAST search on NCBI correspond to some of these SLEs. In fact, except for the element from *A. digitifera* that Metzger et al. [31] found in a closely related group of the Steamer elements, the other sequences are indeed outside of the Steamer family. Although they are phylogenetically close, the fish elements do appear to form an independent group (bootstrap of 32 not shown in Fig. 6); the element of *Branchiostoma belcheri* common to both phylogenies (XM_019773121) is part of the C10 branch, and that of *Strongylocentrotus purpuratus* (XM_011662828) is part of a different branch. This data indicates that the sequences referred to as SLEs seem to correspond more to the whole or part of the Gypsy retrotransposons of the C-clade. In addition, Metzger et al. (2018) [31] point out that many of their SLE sequences have been annotated as K02A2.6-like, based on a more distantly related *Caenorhabditis elegans* retrotransposon. As we note the same in our NCBI searches, it appears that such annotation and element may also be related to the C-clade. Concerning the bivalve SLEs, they were found either directly if their sequences were available in the assembled genomes, or through the sequence of a *C. gigas* SLE that clustered with them with a bootstrap of 100 in the phylogeny of Metzger et al. (2018) [31]. Indeed, it should be noted that the authors use alignments based on sequences between the end of RnaseH and the beginning of Integrase (RT-IN region); whereas our alignments cover the RT/RnaseH domain, which is twice as long (400 vs 200 AA) and known to be more conserved. Several of these bivalve SLEs stand out in branches other than the Steamer family (‘Cerasoderma edule 3’ is part of the C11 branch; ‘*Mercenaria mercenaria* 1’ belongs to C4m, ‘*Mytilus trossulus*’ to C15, ‘*Limaria pellucida* 1’ to C5, and ‘Cerasoderma edule 4’ to C3). But the SLEs ‘*Crassostrea virginica*’, ‘*Mercenaria mercenaria* 2’ (which corresponds to our SteMemer) and ‘Cerasoderma edule 1’ do belong to the Steamer family. This seems to confirm that in addition to the 19 species we obtained, the Steamer family is also represented in *Ishadium recurvum*, *Siliqua patula*, *Limecola balthica* and *Panepoa generosa*.

The distribution of the Steamer family within species is quite peculiar. Although almost exclusively restricted to bivalves, it could not be detected in 6 of the genomes studied. Of course, the absence of detection does not mean absence of element even if we tried to be as exhaustive as possible. Indeed, in addition to the simple LTRHavest search, we used both a RepeatMasker approach and a BLAST search on public databases (using the identified Steamer sequences) in order to either detect a possible Steamer element in apparently devoid species (which allowed us to identify the deleted copies in *R. philippinarum*, and *C. virginica*), or to detect other possible families in species with a Steamer element (which allowed us to reveal the SteMygal2 family). The SteMygal2 sequence (GHIK01193188) comes from transcriptomic data on an individual from Croatia. It is therefore possible that it may not be detected in the genome assembly of an individual collected from the Atlantic Ocean particularly in light of the reported gene presence/absence variations in the species [44], especially in the case of highly mobile features such as transposable elements. Surprisingly, this population variation seems to be weak in *M. mercenaria* since the two American and YKG strains have very close numbers of copies. The difficulty of detection is also partly related to that of the characterization of the Steamer elements with respect to the other retrotransposons of the C-clades to which they remain very close. For instance, the elements of the C9 and C10 branches share the same features in terms of size, length/beginning/end of LTRs, and primer-binding site, even though no Steamer signature could be found in the conserved sites of the coding domains. Sequences obtained by BLAST search from Steamer sequences can actually belong to another branch, and the other way around. In fact, we have not been able to establish diagnostic sequence similarity to simply identify these elements. Therefore, it seems that only a phylogenetic tree approach can distinguish Steamer retrotransposons from other Gypsy retrotransposons.

But especially Steamer elements are difficult to detect because this group seems to be relatively rare. Some species have only deleted copies, and in others the number of full-size copies is very limited and thus the number of copies potentially able to transpose is even smaller. Thus, only 6 families have at least one copy with an intact single ORF (including *M. mercenaria*), which is not the case for any of the 7 families from mussels. Only the ark clam *A. broughtonii* presents a few more Steamer elements with two possibly active families out of three. However, in the absence of data on other species, it is not possible to know if this relative abundance is a particularity of the species or is more general among the ark clams. Therefore, no link can be established between Steamer copy number and host phylogeny or genome size. This scarcity of the Steamer retrotransposons is consistent with the presence of low copy numbers of elements in healthy *M. arenaria* (3–10 copies, [23]) and *Cerasoderma edule* (3–6 copies, [20]). It is also consistent with the patchy distribution of Steamer elements in host species or populations. These elements appear to have weak dynamics and may only be maintained by rare transposition events related to environmental stresses; as when temperature and pH variations significantly induce Steamer expression in juvenile soft-shell clams [76]. It is also possible that the abundance of Steamer elements is strongly controlled, because they are highly expressed and amplified to high copy number in neoplastic cells (with a DNA copy number massively amplified to 150–300 copies in *M. arenaria*, [23]).

Overall, the number of copies of Steamer retrotransposons identified in *M. mercenaria* genomes is higher than in other members of the Venerida order sequenced so far. This suggests that these elements have been recently, and are potentially still, active. However, whatever the species considered, the number of Steamer copies estimated remains much lower than the hundreds of copies detected by qPCR or Southern blotting in leukemic hemocytes of *M. arenaria* [23]. This finding reinforces the link between Steamer elements and leukemia, although, as already pointed out by the authors, it remains unclear if Steamer activation is a consequence or a cause of tumor development. Thus, *M. mercenaria* will represent a prime target for future investigation of retrotransposon dynamics during neoplasia development.

## Conclusions

A chromosome-level assembly of the hard clam genome has been produced and was compared to that of other bivalve species. Results showed peculiar characteristics of the *M. mercenaria* genome, including chromosome rearrangement within the same species, a low percentage of coding regions, and a marked expansion in genes involved in microbe recognition and binding, apoptosis regulation and pro-inflammatory processes, all of which are hallmark of invertebrate innate immunity. A characterization of transposable elements in the hard clam was also performed. Given the increasing reports of disseminated neoplasia in the hard clam, the identification of Steamer and other transposable elements in our genome provides molecular targets for future investigations focusing on carcinogenesis and neoplasia development (and potential transmission) in this species.

## Methods

We used the high-performance computing server (Bridges) of the Extreme Science and Engineering Discovery Environment (XSEDE) to perform all bioinformatic analyses (supported by National Science Foundation grant number ACI-1548562) [77].

### Animal collection and genome sequencing

The genome of *M. mercenaria* comes from an adult clam bred and grown at the Frank M. Flower and Sons Oyster Company in Oyster Bay, New York. DNA was extracted from the adductor muscle using phenol-chloroform method [11]. High molecular weight DNA was submitted to sequencing as described below.

Initial sequencing effort allowed the generation of Illumina Hiseq PE150 (~85x coverage) and Pacbio Sequel I (~20x coverage) reads described elsewhere [11]. In addition to this previously described work, we performed additional sequencing using Pacbio Sequel II and Hi-C technologies to generate more sequence information from the same individual adult hard clam. Purified high molecular weight gDNA was prepped for PacBio single-molecule real-time (SMRT) sequencing using the Express Template Preparation Kit 2.0 (Pacific Biosciences) and following the manufacturer’s instructions. Briefly, 2 μg of gDNA was sheared to generate 10 kb libraries using Covaris g-TUBEs and then concentrated with 0.45X AMPure PB beads (Pacific Biosciences). The sheared gDNA was enzymatically treated to remove single-strand overhangs and repair nicked DNA templates, followed by an End Repair and A-tailing reaction to repair blunt ends and polyadenylate each template. Next, overhang SMRTbell adapters were ligated onto each template and purified using 0.45X AMPure PB beads to remove small fragments and excess reagents. The purified SMRTbell libraries were then size selected at 6–50 kb using the BluePippin system on 0.75% agarose cassettes and S1 ladder, as specified by the manufacturer (Sage Science). The final size-selected library was then annealed to sequencing primer v4 and bound to sequencing polymerase 1.0 before being sequenced on two 8 M SMRTcells on the Sequel II system, each with a 20-h movie, yielding a total of 17,035,649 reads (110X) with a mean length of 38,828b. In addition, an aliquot sample of adductor muscle was immediately frozen in liquid nitrogen before used for Hi-C library preparation with an Arima Genomics Hi-C kit (San Diego, CA, USA) using manufacturer’s instruction. The Hi-C library was then sequenced on one lane of an Illumina HiSeqX PE150 at the Genome Quebec Innovation Center (Mc Gill University). A total of 467,806,558 paired-end reads were generated.

### Genome assembly

A first assembly was generated using sequences derived from all the PacBio data. Following the strategies recommended by Guiglielmoni et al. [78], wtdbg2 assembler [79] was used with default parameters generating a 2Gb-size genome, then we corrected possible haploid contigs using purge_haplotigs [80]. The last step of this first assembly was the polishing using HyPo [81] with the short-read sequences. Finally, this assembly was improved thanks to Hi-C data. Briefly, the Hi-C reads were processed using hicstuff [82] with the parameters --enzyme DpnII,HinfI --iterative. The pipeline includes a mapping step against the contigs using bowtie2 [83]. Then, instaGRAAL [84] was run with the parameters --level 5 --cycles 100 --coverage-std 1 --neighborhood 5, and the output was further improved with instagraal-polish. Based on the universal single-copy orthologs (BUSCO) analysis using the Mollusca_odb10 and Metazoa_odb10 lineages, we assessed the quality of the final assembly and contrasted it to other bivalve genome assemblies. Blobtools [85] was run with default parameters on the final assembly of the clam in order to detect potential contamination. For that, reads from Illumina were mapped on the assembly using BWA mem algorithm [86] and BLASTn version 2.11.0 [87] was also computed on the NT database from NCBI [88] and given as input to blobtools.

In order to compare our assembly and *M. mercenaria* YKG genome assembly, nucmer from MUMmer [89] was run with default parameters comparing each chromosome from one strain to all chromosomes from the second strain. Mummerplot was used to generate dot plots of the results.

### LTR-retrotransposons and steamer elements identification

We investigated LTR-retrotranspons using a detailed and precise pipeline customized for *M. mercenaria.* This was done because we were interested in the characterization of Steamer elements (part of the C-clade of the Gypsy superfamily) in Bivalvia, given the suspected role of these elements in carcinogenesis and the increasing reports of leukemia in the hard clam. Thus, we first choose to refine the detection of LTR-retrotransposons by running LTRHarvest [90] on all Bivalvia genomes using the following parameters “-minlenltr 80 -maxlenltr 1200 -mindistltr 2500 -maxdistltr 11000 -similar 80.0”. The outputs were combined and filtered using BLASTx [87] (evalue less than 10^− 15^) against an in-house database (267 RT/RNaseH or *pol* sequences representing the different known clades of LTR-retrotransposons, plus DIRS and Polinton sequences used as competitors) assigning, when possible, the resulting sequences to each LTR-superfamily (Gypsy, Copia or BEL/Pao) if more than 8 of the first ten matches were assigned to the same superfamily; uncertain assignation were manually curated. Sequences belonging to each superfamily were clustered into families using uclust from USEARCH version 11.0.667 [91] with parameter “-cluster_fast -id 0.8 -sort length -strand both”. A multiple alignment was done on each cluster of sequences and on the remaining single sequences (clustered together by species). Then sequences were inserts-cleaned using an in-house program trimming the nucleotides not conserved in at least 80% of the aligned sequences. This pipeline (from uclust to trimming) was performed twice to get a better clustering.

To identify Steamer elements, we first extracted and translated the RT/RNaseH domain from the Gypsy sequences obtained with LTRHarvest [90] for all 20 species. This was done using BLASTx [87] (E-value less than 10E-5) against an in-house database of RT/RNaseH of 215 Gypsy elements representing a large part of the Gypsy clades [32]; best matches positions guided the extraction with boundaries of RT/RNaseH domains being determined according to those defined for RT 5′ part and RNaseH 3′ part of Gypsy multiple alignments defined in the Gypsy Database [92]. This Gypsy dataset (including *Steamer* elements from *M. arenaria* and *E. directus*, AIE48224.1 and MH025794, respectively, [31]) was used as database to retrieve potential Gypsy elements from the C-clade using BLASTp [87] and keeping sequences having the best match with C-clade reference elements with an E-value less than 10E-50 and at least 300aa covered. Here, we kept the single sequences and a consensus sequence per previously defined-cluster. To identify Steamer elements more widely in metazoans, we performed tBLASTn [87] analyses with the RT/RNaseH domain of the *Steamer* element from *M. arenaria* as query (E-values 1E-140, query cover > 95%, no filter) on genomic and transcriptomic databases (nr/nt, wgs, est., TSA) available at NCBI (https://www.ncbi.nlm.nih.gov/).

We also used phylogenetic approaches to determine the position of the C-clade elements in each branch. Phylogenetic analyses were performed as in Thomas-Bulle et al. [32] on amino acid sequences corresponding to the RT/RNaseH domains of the newly characterized sequences and reference elements from the C-clade. Multiple alignments of these protein sequences were performed using MAFFT [93]. After a manual curation of the alignments, phylogenetic analyses were conducted using Neighbor Joining and the pairwise deletion option of the MEGA5.2 software [94]. Using Topali2.3 [95], the best-fitted substitution model retained was the JTT model with a gamma distribution. Support for individual groups was evaluated with non-parametric bootstrapping using 100 replicates.

Finally, we used a RepeatMasker version 4.1.0 [96] approach with the addition of “Concatenate_sequences.py” concatenating hits closer than 500 bp and removing hits smaller than 300 bp length [32] to (i) search Steamer elements in bivalve genome devoid of a complete copy, and (ii) retrieve all possible Steamer copies in each genome. In the first case, all Steamer copies identified so far, whatever the species considered, were used as input to RepeatMasker [96]. In the second case, only Steamer sequences from the considered species were used as a library for RepeatMasker [96].

### Repeated sequences annotation

Repeated sequences were annotated in both *M. mercenaria* genomes (from this study and YKG) by running RepeatMasker [96] with default parameter and using different libraries at different steps of the annotation. (i) The first step was done to detect potential satellites, previously identified in *M. mercenaria* and available on NCBI (EU380194.2-EU380201.2, KR704602.1-KR704618.1, GQ121374.1-GQ11407.1, GQ397363.1, GQ397364.1 and AF108910.1- AF108912.1, AF108921.1-AF108943.1, unpublished). This step included the identification of all satellites, micro-satellites, simple repeats and low complexity sequences by masking them in both genomes. (ii) The second step consisted in annotating and masking the previously predicted LTR-retrotransposons detected within the clam genome by LTRHarvest [90]. (iii) These sequences were then clustered using uclust (“-cluster_fast -id 0.8 -sort length -strand both, [91]) and inserts were removed within each cluster. This procedure was done twice. Then, only consensus from each cluster was given as a library for RepeatMasker [96] to retrieve putatively missed LTR-retrotransposon copies (with corrupted LTRs or deleted) on the rest of the genomes. (iv) The last step aimed to use RepeatModeler v2.0.1 [97] (using REPBASE, version 2017-01-27, [98]) generating the library for RepeatMasker [96] to complete the annotation of all other transposable element types. “Concatenate_sequences.py” [32] was used in (ii, iii and iv) concatenating hits closer than 500 bp and removing hits smaller than 300 bp in length.

### Genome annotation

A previously published transcriptome [9] of *M. mercenaria*, predicted genes from Song et al. [24], proteins belonging to Mollusca and reviewed sequences belonging to Bilateria species from UniProt database [99] were mapped on the repeat-masked genome with BLAT [100] to rapidly identify the position of the sequences on the genome. To refine the alignments, only matches with more than 80% identity were kept and given to exonerate version 2.4.0 using est2genome model and protein2genome model for transcriptome and proteins mapping, respectively. Transcriptome alignments were filtered with at least 98% of identity and at least 90% of the transcript length matching the genome while proteins hits were filtered with at least 50% of identity and at least 50% of the protein length matching the genome. An ab-initio prediction was done using SNAP [101] with training on the transcriptome sequences mappings. Finally, Gmove [102] combined all different resources listed above with the addition of the previous gene prediction to build the gene set. Finally, this gene set was given to Gmove [102] with the addition of the mapped predicted annotation from Song et al. [24]. Functional annotation was done on each resulting protein by alignments to the nr database [103] using BLASTp version 2.11.0 [87] by keeping the best three matches. Domains were defined using InterProScan 5.36–75.0 [104] with the default parameters. Finally, correspondence was done between InterProScan identifications and gene ontology terms [105, 106]. The assessment of the predicted proteins was done based on BUSCO by mapping the Metazoa_obd10 database [107] on all considered assemblies. The script “agat_sp_statistics.pl” from *A*nother *G*tf/Gff *A*nalysis Toolkit (AGAT) was run against all genomes and related GFF files to compute all annotation metrics.

In order to compare annotations between *M. mercenaria* and *M. mercenaria* YKG gene prediction, Orthofinder version 2.4.1 [28] was ran using the two proteomes with default parameters. In addition, Best Reciprocal Hits (BRH) were determined to retrieve 2 by 2 orthologs using BLASTp matches filtered on an E-value of 10E-5. Moreover, GO terms were assigned to each gene from each assembly using IPR2GO database and TopGO library from R was used to generate statistics of GO enrichment in genes present in one strain but not found in the other one and conversely.

### Gene duplications and gene family analysis on 20 Mollusca species

Duplication events were assessed by running MCScanX [108] with default parameters and as input the BLASTp file result of all proteins predicted against each other and the *M. mercenaria* genome sequences. We also ran ‘duplicate_gene_classifier’, in order to reveal all different type of duplications detected in our genome assembly. GO terms were assigned to each genes using IPR2GO database. Then, TopGO R library was used to generate statistics of GO enrichment in different categories of genes (e.g., tandem duplicated genes).

Predicted *M. mercenaria* proteome was compared to the newly published hard clam genome YKG (GCA_014805675.1, [24]) as well as to other published genomes of Bivalvia representing 6 orders including Venerida with *R. philippinarum* (GCA_014805675.1, [37]), *C. sinensis* (GCA_012932295.1, [35]), *L. rhynchaena* (GCA_008271625.1, [36]) and *A. marissinica* (GCA_014843695.1, [34]), Myida with *Dreissena rostriformis* (GCA_007657795.1, [109]), Adapedonta with *Sinonovacula constricta* (GCA_007657795.1, [110]), Arcida with *A. broughtonii* (no accession number, [111]), Ostreida with *C. gigas* (GCA_902806645.1, [112]), *C. virginica* (GCA_002022765.4, [113]), *O. edulis* (unpublished), *S. glomerata* (GCA_003671525.1, [114]), Pectinida with *Mizuhopecten yessoensis* (GCA_002113885.2, [115]) and *P. maximus* (GCA_902652985.1, [116]) and Mytilida with *M. philippinarum* (GCA_002080025.1, [117]), *Limnoperna fortunei* (GCA_003130415.1, [118]), *M. coruscus* (GCA_011752425.2, [119]) and *M. galloprovincialis* (GCA_900618805.1, [44]) with the addition of an outgroup species from the Gastrodopa class, *Aplysia californica* (GCA_000002075.2, [120]). These Predicted proteomes were downloaded from public databases or requested from authors if not public. *A. californica* was used as outgroup. In order to define gene families, Orthofinder [28] was used on all previous proteomes with default parameters (*P. fucata* was removed from the analysis as it had less than 80% of the proteome within orthogroups). From Orthofinder results, we used the single copy gene OGs and concatenated the proteins per species to generate the species tree using MAFFT online [93] with default parameters adding the bootstrap calculation and using iTOL to generate a graphical representation of the tree.

TNF and C1q domain-containing genes belonging to *M. mercenaria* were manually identified and curated. Briefly, the identification was first based on the presence of the domain of interest, with the second step being the retrieval of all genes being in the same OG. Then, genes not having a start or end codon or having a deletion region were verified and corrected where possible, using IGV [121] with RNAseq data for validation using splicing sites and mapped proteic and transcriptomic data that served for the annotation process. Once validated, all domains of interest were extracted from all considered species using an in-house script. Multiple alignments of all retrieved domains (444 and 2217 sequences of TNF and C1q domain, respectively) were done with the MAFFT server using MaxAlign tool (293 and 408 curated sequences left for TNF and C1q domains respectively) to improve the alignment and run a phylogeny with bootstrap of 1000 on a Neighbor Joining method. Trees were generated using iTOL.

## Supplementary Information

**Additional file 1: Figure S1**: Taxonomy assignation of the contigs in the assembly. **Figure S2**: Ortholog identity percentages. **Figure S3**: Number of copy differences between both strains of *M. mercenaria*. **Figure S4**: Number of ortholog genes shared by selected species. **Figure S5**: Distribution and duplication types of c1q domains among *M. mercenaria* chromosomes. **Figure S6**: Phylogenetic tree of the 18 orthogroups that contained 50% of the C1q domains from *M. mercenaria*. **Figure S7**: Alignment of C1q domains from consensus sequences per orthogroups in *M. mercenaria*. **Figure S8**: Phylogenetic relationships of bivalves Gypsy retrotransposons from the C-clade. **Table S1**: Bivalvia assembly metrics. **Table S2**: Gene annotation metrics of Bivalvia genomes. **Table S3**: Comparison between both *M. mercenaria* genome assemblies. **Table S9**: TNF-domain containing orthogroups. **Table S10**: Comparison between structural features of Steamer retrotransposons.

**Additional file 2: Table S4**: GO enrichment analysis. The enrichment analysis, using topGo, of genes identified in *M. mercenaria* in this study but not found in *M. mercenaria YKG* (3 first columns) and genes found in *M. mercenaria YKG* but not in our *M. mercenaria* assembly (3 last columns). **Table S5**: Functional annotation and gene duplication. For each gene annotated in this study, gene duplication events (0: “Not duplicated” 1:"dispersed duplication” 2:"proximal duplication” 3:"tandem duplication” 4:"segmental duplication”), Gene Ontology identification and the three first best matches on NR are listed. **Table S6**: GO enrichment in duplicated genes. GO enrichment analysis using topGO was performed on the genes found duplicated within *M. mercenaria* genome. Results were sorted by *p* value. **Table S7**: Genes expanded in *M. mercenaria* across Bivalvia. Orthogroups gene counts across 20 genomes of Bivalvia with expanded genes in *M. mercenaria*. Ave being the mean of copies in each species. Expansion column: 1: genes expanded in *M. mercenaria* not detected in all other species. 2: Genes with copy numbers higher in *M. mercenaria* than the mean number of copies in all other species. 3: Genes with copy numbers higher in *M. mercenaria* than the mean number of copies in all Venerida species. **Table S8**: TNF containing domain genes. List of all *M. mercenaria* genes found in orthogroups containing TNF domains. The CDS start position, the type of duplication (0: “Not duplicated” 1:"dispersed duplication” 2:"proximal duplication” 3:"tandem duplication” 4:"segmental duplication”), the orthogroup ID, the best three first matches on NR, the principal and the number of transmembrane domain and the protein length are reported. Colors represent orthogroups used for the phylogenetic tree (Fig. 4).

## Data Availability

This Whole Genome Shotgun project has been deposited at DDBJ/ENA/GenBank under the accession number JAIXLV000000000. The raw reads for the genome were submitted under BioProject PRJNA638823.

## Abbreviations

C1qDC: C1q domain-containing
LTR: Long terminal repeat
NOR: Nucleolar organizer region
OG: Orthogroup
QPX: Quahog parasite unknown
RT/RNaseH: Reverse Transcriptase/Ribonuclease-H
TE: Transposable elementsSLE: Steamer-like element
TIR: Toll/interleukin-1 receptor
TM: Transmembrane
TNF: Tumor necrosis factor
