# Supplementary material for: Comparative analysis of the Mercenaria mercenaria genome provides insights into the diversity of transposable elements and immune molecules in bivalve mollusks
Source: BMC Genomics. 2022 Mar 8;23:192. doi: 10.1186/s12864-021-08262-1 (PMC8905726; doi:10.1186/s12864-021-08262-1)
Supplement: Supplementary file 1 — Additional file 1: Figure S1: Taxonomy assignation of the contigs in the assembly. Figure S2: Ortholog identity percentages. Figure S3: Number of copy differences between both strains of M. mercenaria. Figure S4: Number of ortholog genes shared by selected species. Figure S5: Distribution and duplication types of c1q domains among M. mercenaria chromosomes. Figure S6: Phylogenetic tree of the 18 orthogroups that contained 50% of the C1q domains from M. mercenaria. Figure S7: Alignment of C1q domains from consensus sequences per orthogroups in M. mercenaria. Figure S8: Phylogenetic relationships of bivalves Gypsy retrotransposons from the C-clade. Table S1: Bivalvia assembly metrics. Table S2: Gene annotation metrics of Bivalvia genomes. Table S3: Comparison between both M. mercenaria genome assemblies. Table S9: TNF-domain containing orthogroups. Table S10: Comparison between structural features of Steamer retrotransposons. [file 12864_2021_8262_MOESM1_ESM.docx]

1. **Supplemental Figures**

**Figure S1: Taxonomy assignation of the contigs in the assembly.**

**
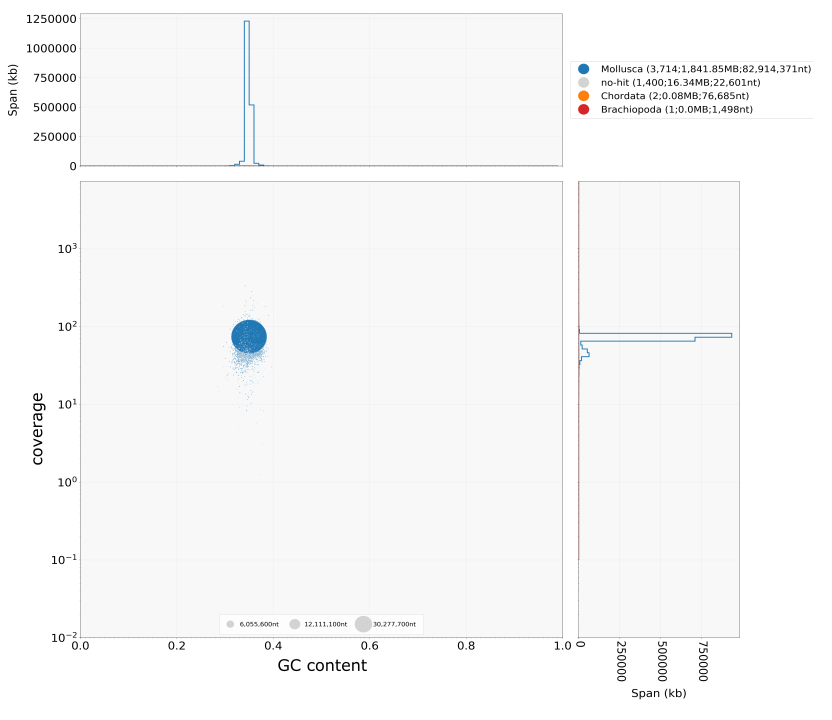
**

Two-dimensional scatter plots generated using Blobtools (86) showing GC content and read coverage histograms. Sequences are represented by circles where the diameter is proportional to the sequence length and the color represents the taxonomic affiliation. Coverage and GC histograms are drawn for each taxonomic group, which are weighted by the total span (cumulative length) of sequences. The legend reflects the taxonomic affiliation of sequences and lists count, total span and N50 by taxonomic group.

**Figure S2: Ortholog identity percentages.**


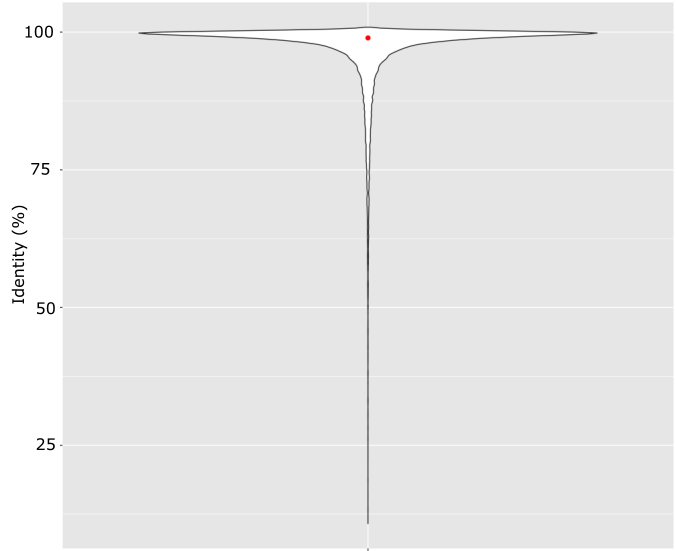


Violin plot of the identity percent between ortholog pairs from our new *Mercenaria mercenaria* and the *M. mercenaria* YKG assembly. Red dot represents the median identity percent.

**Figure S3:** **Number of copy differences between both strains of *M. mercenaria*.**


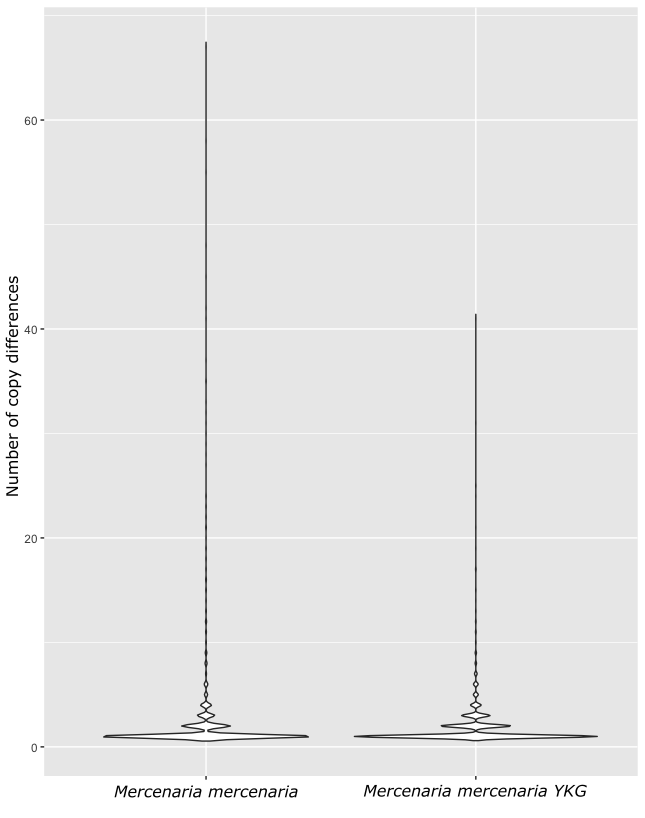


Violin plot of copy differences for each defined OG found having genes belonging to both strains of *M. mercenaria*. Left, number of copy differences in OG having more copies in our *M. mercenaria* genome as compared to YKG. Right, number of copy differences in OG having more copies in *M. mercenaria* YKG (24) as compared to our assembly.

**Figure S4: Number of ortholog genes shared by selected species.**

**
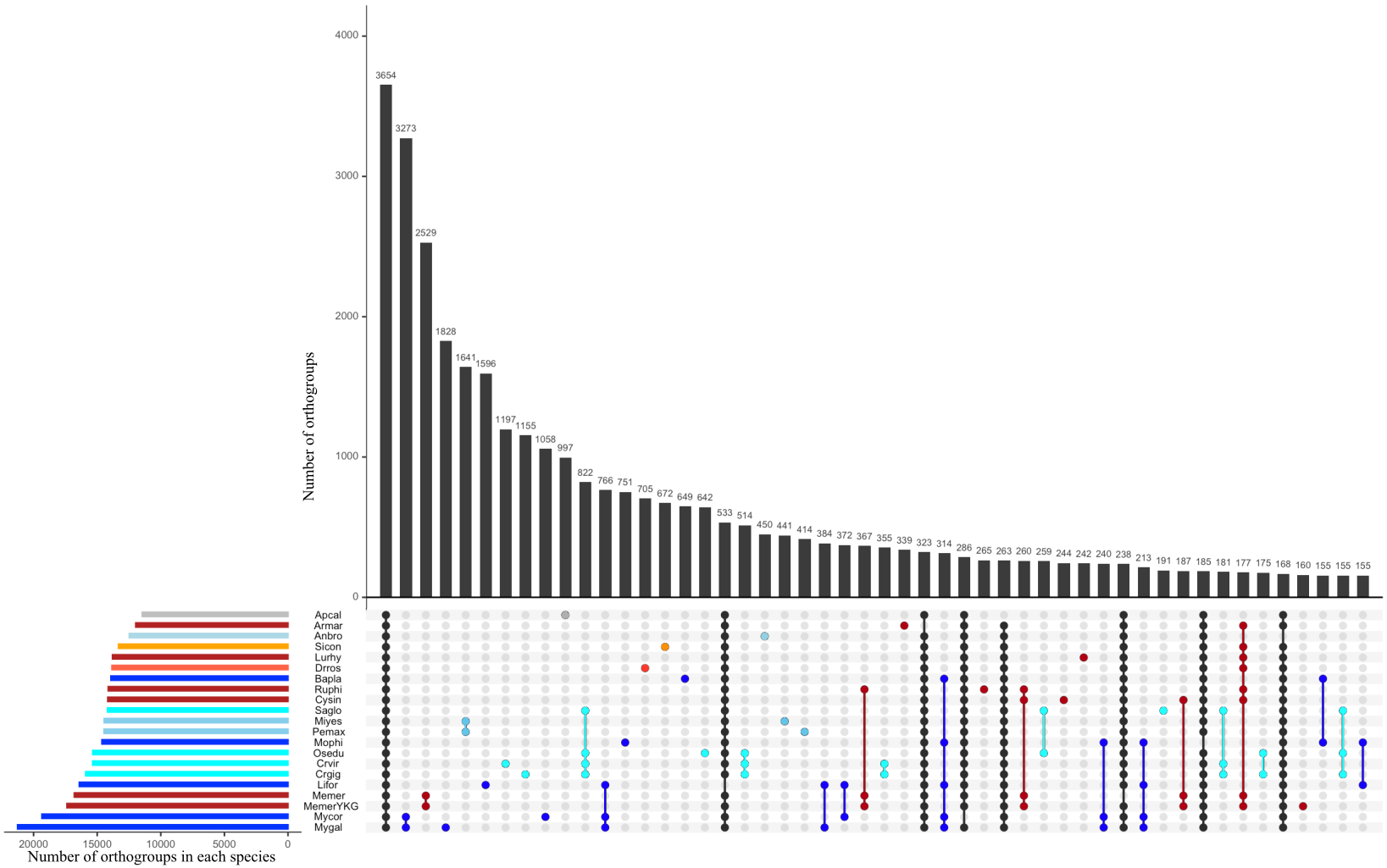
**

Barplot showing the number of clusters having at least one gene copy from each represented species marked by the dots. Left barplot represents the total number of orthogroup per species. Red dots and bars for Venerida order including Armar: *Archivesica marissinica*; Cysin: *Cyclina sinensis*; Lurhy: *Lutraria rhynchaena*; Memer: *Mercenaria mercenaria* and Memer YKG: *Mercenaria mercenaria YKG*; Ruphi: *Ruditapes philippinarum*. Orange for Cardiida order with Sicon: *Sinonovacula constricta*. Light blue for Arcoida order with Anbro: *Anadara broughtonii*. Tomato for Myida order with Drros: *Dreissena rostriformis*. Cyan for Ostreida order including Crgig: *Crassostrea gigas*; Crvir: *Crassostrea virginica*; Osedu: *Ostrea edulis*; Saglo: *Saccostrea glomerata*. Skyblue for Pectinida order including Miyes: *Mizuhopecten yessoensis*; Pemax: *Pecten maximus*. Blue for Mytiloida order including Bapla: *Bathymodiolus platifrons*; Mophi: *Modiolus philippinarum*; Lifor: *Limnoperna fortunei*; Mycor: *Mytilus coruscus*; Mygal: *Mytilus galloprovincialis*. And grey for Aplysiida order (outgroup) Apcal: *Aplysia californica*.

**Figure S5: Distribution and duplication types of c1q domains among *M. mercenaria* chromosomes.**

**
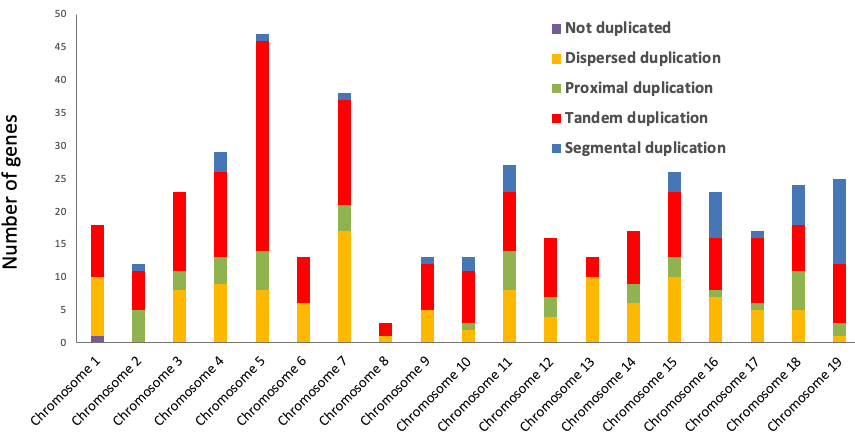
**

Number of C1q domains found on the *M. mercenaria* chromosomes and type of duplication defined using MCScanX (109).

**Figure S6: Phylogenetic tree of the 18 orthogroups that contained 50% of the C1q domains from *M. mercenaria*.**

**
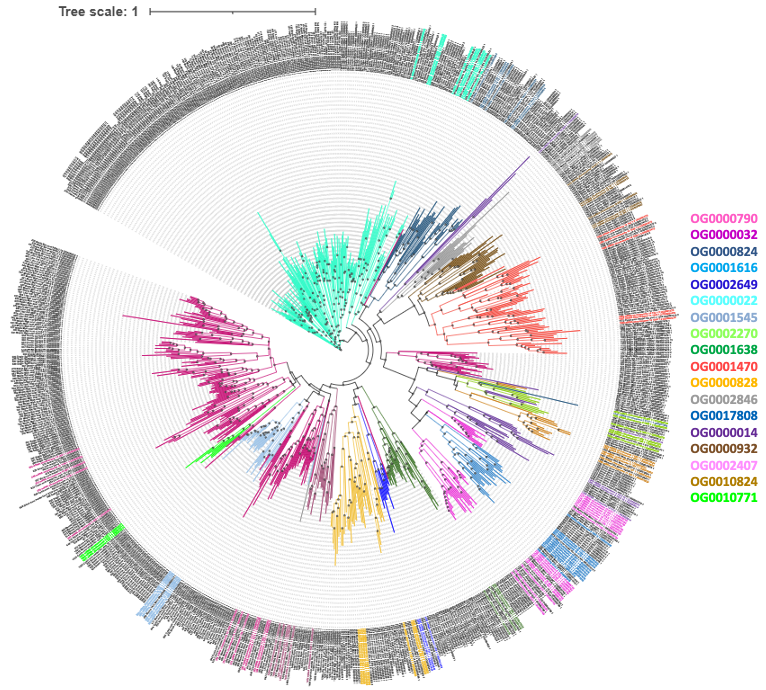
**

Results of 826 sequences of C1q domains after filtering using CDhit followed by MaxAlign from MAFFT (94). Each orthogroup and each *M. mercenaria* sequence label (from this study) is defined by a color.

**Figure S7: Alignment of C1q domains from consensus sequences per orthogroups in *M. mercenaria*.**

**
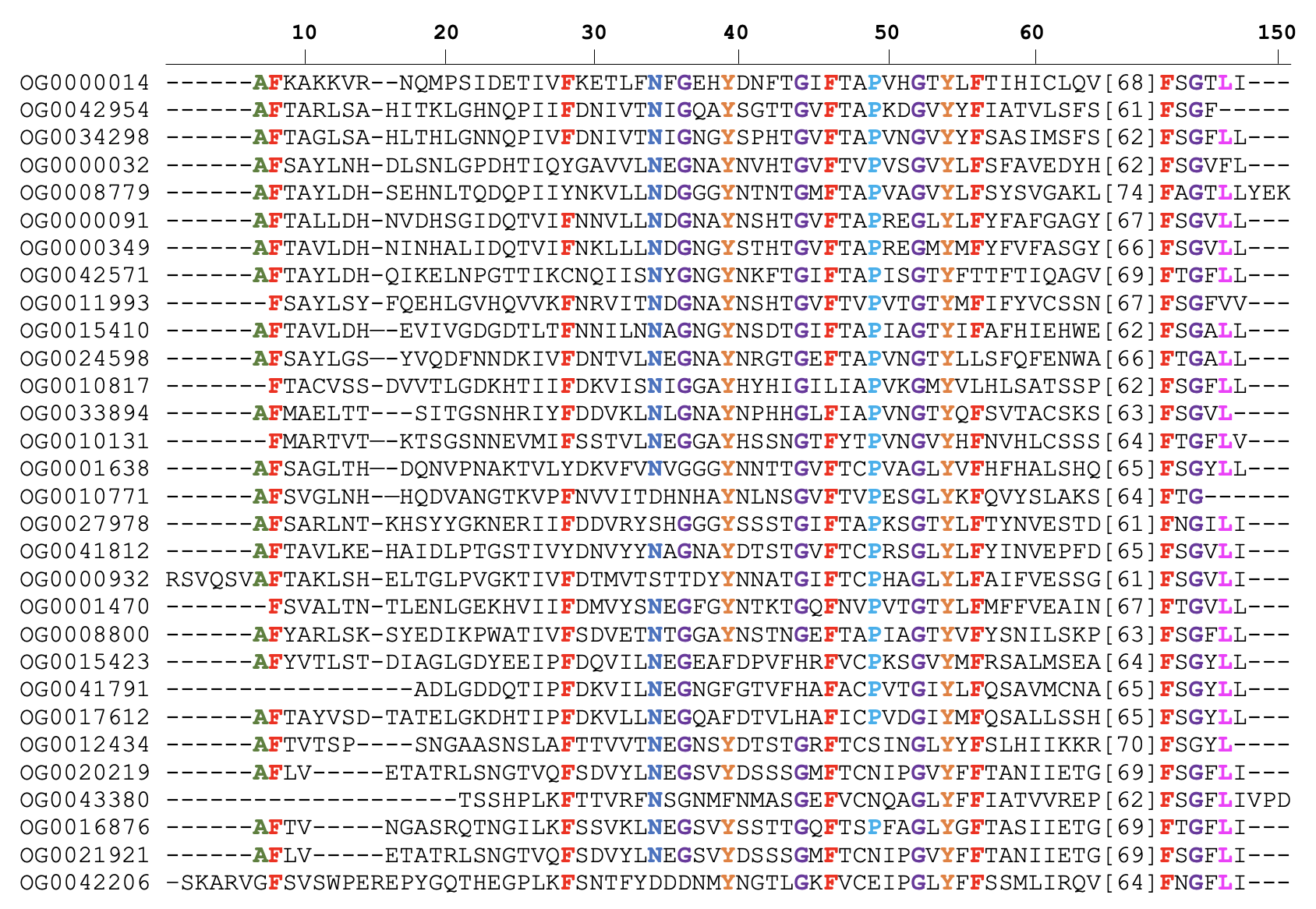
**

A selection of 30 consensus sequences after alignment in MAFFT, followed by a dataset refinement using CD-HIT and MaxAlign. A portion of the sequence within the bracket is not shown.

**Figure S8: Phylogenetic relationships of bivalves Gypsy retrotransposons from the C-clade.**

**
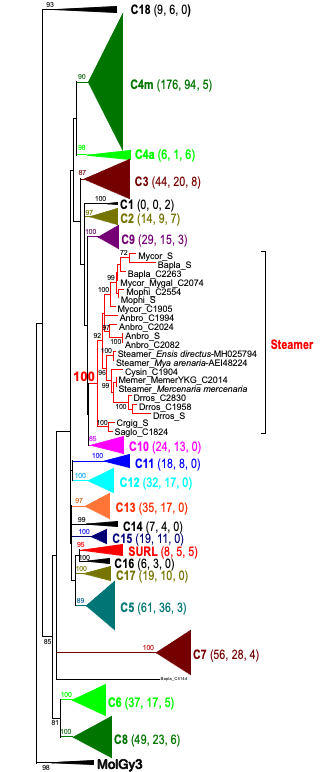
**

This tree is based on Neighbor-Joining analysis (123) of RT/RNaseH domain amino acid sequences with six elements from de MolGy3 (27) clade used as outgroup. Node statistical support values come from non-parametric bootstrapping using 100 replicates and only those >80 % are shown. The C-clade branches are indicated in color on the condensed tree and only the Steamer group is detailed with single sequences indicated with a S and consensus sequences with a C followed by the number of the cluster they represent. Numbers between parentheses represent the number of sequences, clusters, and external references used, respectively. Mycor: *Mytilus coruscus*; Mygal: *Mytilus galloprovincialis*; Bapla: *Bathymodiolus platifrons*; Mophi: *Modiolus philippinarum*; Anbro: *Anadara broughtonii*; Cysin: *Cyclina sinensis*; Memer: *Mercenaria mercenaria*; YKG: *Mercenaria mercenaria YKG*; Drros: *Dreissena rostriformis*; Crgig: *Crassostrea gigas*; Saglo: *Saccostrea glomerata*.

1. **Supplemental tables**

**Table S1: Bivalvia assembly metrics.**

| Clade | Order | Family | Genome accession number | Specie | Genome size | Number of sequences | N50 | GC % | BUSCO (mollusca_odb10) | BUSCO (metazoa_odb10) |
| --- | --- | --- | --- | --- | --- | --- | --- | --- | --- | --- |
| Heteroconchia | Venerida | **Veneroidae** | **This article** | ***Mercenaria mercenaria**** | **1.86Gb** | **5121 (19 chromosomes)** | **83Mb** | **35%** | **C:76.4%[S:73.4%,D:3.0%],**  **F:4.7%,M:18.9%,n:5295** | **C:91.8%[S:86.2%,D:5.6%],**  **F:4.0%,M:4.2%,n:954** |
| Heteroconchia | Venerida | Veneroidae | GCA_014805675.1 | *Mercenaria mercenaria YKG** | 1.79Gb | 1537 (19 chromosomes) | 91Mb | 35% | C:74.7%[S:72.5%,D:2.2%],  F:5.1%,M:20.2%,n:5295 | C:91.2%[S:87.2%,D:4.0%],  F:3.1%,M:5.7%,n:954 |
| Heteroconchia | Venerida | Veneroidae | [GCA_009026015.1](https://www-ncbi-nlm-nih-gov.proxy.library.stonybrook.edu/assembly/4887211) | *Ruditapes philippinarum** | 1.1Gb | 26963 (19 chromosomes) | 46.3Mb | 32% | C:76.1%[S:74.5%,D:1.6%],  F:5.5%,M:18.4%,n:5295 | C:90.8%[S:89.1%,D:1.7%],  F:5.6%,M:3.6%,n:954 |
| Heteroconchia | Venerida | Veneroidae | GCA_012932295.1 | *Cyclina sinensis** | 903.2Mb | 187 (19 chromosomes) | 46.5Mb | 34% | C:78.3%[S:72.4%,D:5.9%],  F:4.9%,M:16.8%,n:5295 | C:92.7%[S:84.9%,D:7.8%],  F:3.5%,M:3.8%,n:954 |
| Heteroconchia | Venerida | Vesicomyidae | GCA_008271625.1 | *Archivesica marissinica** | 1.5Gb | 4,005 (19 chromosomes) | 74Mb | 39% | C:72.9%[S:71.9%,D:1.0%],  F:5.1%,M:22.0%,n:5295 | C:89.8%[S:88.1%,D:1.7%],  F:6.3%,M:3.9%,n:954 |
| Heteroconchia | Venerida | Mactridae | GCA_014843695.1 | *Lutraria rhynchaena* | 544Mb | 622 scaffolds | 2Mb | 34% | C:80.3%[S:78.9%,D:1.4%],  F:4.5%,M:15.2%,n:5295 | C:94.5%[S:93.0%,D:1.5%],  F:3.7%,M:1.8%,n:954 |
| Heteroconchia | Myida | Dreissenidae | GCA_007657795.1 | *Dreissena rostriformis* | 1.2Gb | 18,514 scaffolds | 131Kb | 35% | C:55.6%[S:53.2%,D:2.4%],  F:6.7%,M:37.7%,n:5295 | C:74.9%[S:72.4%,D:2.5%],  F:15.7%,M:9.4%,n:954 |
| Heteroconchia | Cardiida | Solecurtidae | GCA_007844125.1 | *Sinonovacula constricta** | 1.2Gb | 362 (19 chromosomes) | 66Mb | 35% | C:76.4%[S:69.8%,D:6.6%],  F:4.8%,M:18.8%,n:5295 | C:91.5%[S:80.5%,D:11.0%],  F:4.0%,M:4.5%,n:954 |
| Pteriomorphia | Arcoida | Arcidae | ND | *Anadara broughtonii** | 885Mb | 1026 (19 chromosomes) | 45Mb | 34% | C:85.6%[S:83.1%,D:2.5%],  F:3.1%,M:11.3%,n:5295 | C:92.1%[S:86.9%,D:5.2%],  F:1.5%,M:6.4%,n:954 |
| Pteriomorphia | Ostreida | Ostreidae | GCA_902806645.1 | *Crassostrea gigas ** | 558Mb | 10 chromosomes | 401.7Kb | 34% | C:98.2%[S:96.2%,D:2.0%],  F:0.5%,M:1.3%,n:5295 | C:98.4%[S:95.4%,D:3.0%],  F:0.7%,M:0.9%,n:954 |
| Pteriomorphia | Ostreida | Ostreidae | GCA_002022765.4 | *Crassostrea virginica** | 685Mb | 10 chromosomes | 75.9Mb | 35% | C:97.4%[S:85.4%,D:12.0%],  F:0.7%,M:1.9%,n:5295 | C:98.2%[S:80.6%,D:17.6%],  F:0.2%,M:1.6%,n:954 |
| Pteriomorphia | Ostreida | Ostreidae | unpublished | *Ostrea edulis* | 872Mb | 10 chromosomes | 97.7Mb | 35% | C:93.7%[S:92.4%,D:1.3%],  F:0.9%,M:5.4%,n:5295 | C:94.8%[S:92.9%,D:1.9%],  F:0.7%,M:4.5%,n:954 |
| Pteriomorphia | Ostreida | Ostreidae | GCA_003671525.1 | *Saccostrea glomerata* | 788Mb | 10,101 scaffolds | 804.2Kb | 33% | C:89.4%[S:86.4%,D:3.0%],  F:2.7%,M:7.9%,n:5295 | C:91.6%[S:87.7%,D:3.9%],  F:6.2%,M:2.2%,n:954 |
| Pteriomorphia | Pectinida | Pectinidae | GCA_002113885.2 | *Mizuhopecten yessoensis* | 988Mb | 82,659 scaffolds | 803.6Kb | 37% | C:97.5%[S:96.7%,D:0.8%],  F:0.9%,M:1.6%,n:5295 | C:96.9%[S:94.0%,D:2.9%],  F:2.0%,M:1.1%,n:954 |
| Pteriomorphia | Pectinida | Pectinidae | GCA_902652985.1 | *Pecten maximus** | 918Mb | 3,983 (19 chromosomes) | 45Mb | 37% | C:95.8%[S:94.2%,D:1.6%],  F:1.2%,M:3.0%,n:5295 | C:97.1%[S:93.9%,D:3.2%],  F:1.2%,M:1.7%,n:954 |
| Pteriomorphia | Mytiloida | Mytilidae | GCA_002080005.1 | *Bathymodiolus platifrons* | 1.66Gb | 65,662 scaffolds | 343.4Kb | 34% | C:83.3%[S:82.0%,D:1.3%],  F:4.7%,M:12.0%,n:5295 | C:92.7%[S:91.4%,D:1.3%],  F:4.9%,M:2.4%,n:954 |
| Pteriomorphia | Mytiloida | Mytilidae | GCA_002080025.1 | *Modiolus philippinarum* | 2.6Gb | 74,573 scaffolds | 100Kb | 34% | C:73.2%[S:71.2%,D:2.0%],  F:6.3%,M:20.5%,n:5295 | C:87.0%[S:83.9%,D:3.1%],  F:9.4%,M:3.6%,n:954 |
| Pteriomorphia | Mytiloida | Mytilidae | GCA_003130415.1 | *Limnoperna fortunei* | 1.6Gb | 20580 scaffolds | 309Kb | 34% | C:56.0%[S:54.7%,D:1.3%],  F:7.4%,M:36.6%,n:5295 | C:66.8%[S:65.0%,D:1.8%],  F:19.3%,M:13.9%,n:954 |
| Pteriomorphia | Mytiloida | Mytilidae | GCA_011752425.2 | *Mytilus coruscus* | 1.9Gb | 10484 scaffolds | 898Mb | 32% | C:81.9%[S:79.4%,D:2.5%],  F:4.2%,M:13.9%,n:5295 | C:92.2%[S:88.3%,D:3.9%],  F:4.7%,M:3.1%,n:954 |
| Pteriomorphia | Mytiloida | Mytilidae | GCA_900618805.1 | *Mytilus galloprovincialis* | 1.3Gb | 10,577 scaffolds | 207Kb | 32% | C:70.5%[S:65.1%,D:5.4%],  F:4.8%,M:24.7%,n:5295 | C:79.2%[S:71.4%,D:7.8%],  F:9.2%,M:11.6%,n:954 |
| Euthyneura | Aplysiida | Aplysiidae | GCA_000002075.2 | *Aplysia californica* | 927Mb | 4,332 scaffolds | 918Kb | 40% | C:89.7%[S:89.5%,D:0.2%],  F:2.5%,M:7.8%,n:5295 | C:93.3%[S:93.1%,D:0.2%],  F:3.1%,M:3.6%,n:954 |

Global assembly metrics of the *M. mercenaria* genome as compared to other members of the Bivalvia class, with the gastropod *A. californica* as outgroup. Na (not applicable). C: complete, S: Single, D: Duplicated, F: fragmented, M: missing and n: number of sequences in databases. * in species column indicate chromosome-level assemblies.

**Table S2: Gene annotation metrics of Bivalvia genomes.**

| Clade | Order | Family | Specie | Genome size | nb genes (protein coding genes) | Mean CDS length (bp) | Mean Exons length (bp) | Introns length (bp) | Number of exons/cds | CDS coverage (%) | BUSCO on predicted proteins (%) |
| --- | --- | --- | --- | --- | --- | --- | --- | --- | --- | --- | --- |
| Heteroconchia | Venerida | **Veneroidae** | ***Mercenaria mercenaria**** | **1.86Gb** | **34728** | **1113** | **214** | **2474** | **5,5** | **2,1** | **84.7** |
| Heteroconchia | Venerida | Veneroidae | *Mercenaria mercenaria YKG** | 1.79Gb | 34283 | 1219 | 258 | 2844 | 6 | 2,3 | 78.1 |
| Heteroconchia | Venerida | Veneroidae | *Ruditapes philippinarum** | 1.1Gb | 27652 | 1462 | 227 | 1697 | 7,3 | 3,7 | 87.3 |
| Heteroconchia | Venerida | Veneroidae | *Cyclina sinensis** | 903.2Mb | 27564 | 1471 | 278 | 1779 | 7,4 | 4,5 | 94.0 |
| Heteroconchia | Venerida | Vesicomyidae | *Archivesica marissinica** | 1.5Gb | 29209 | 1184 | 280 | 2990 | 4,8 | 2,2 | 82.0 |
| Heteroconchia | Venerida | Mactridae | *Lutraria rhynchaena* | 544Mb | 26380 | 1138 | na | na | na | 5,5 | 82.5 |
| Heteroconchia | Myida | Dreissenidae | *Dreissena rostriformis* | 1.2Gb | 37681 | 1160 | 194 | 1615 | 6 | 3,6 | 75.2 |
| Heteroconchia | Cardiida | Solecurtidae | *Sinonovacula constricta** | 1.2Gb | 28594 | 1484 | 303 | 2485 | 7,6 | 3,5 | 92.5 |
| Pteriomorphia | Arcoida | Arcidae | *Anadara broughtonii** | 885Mb | 24045 | 1595 | 232 | 1617 | 7,6 | 4,3 | 91.2 |
| Pteriomorphia | Ostreida | Ostreidae | *Crassostrea gigas** | 558Mb | 31371 | 2008 | 258 | 1218 | 11,1 | 19,6 | 98.5 |
| Pteriomorphia | Ostreida | Ostreidae | *Crassostrea virginica** | 685Mb | 34608 | 1627 | 262 | 1026 | 8,8 | 8,2 | 98.1 |
| Pteriomorphia | Ostreida | Ostreidae | *Ostrea edulis** | 872Mb | 37386 | 1713 | 327 | 1732 | 7,6 | 8,9 | 91.3 |
| Pteriomorphia | Ostreida | Ostreidae | *Saccostrea glomerata* | 788Mb | 29738 | 1529 | 215 | 964 | 7,8 | 5,8 | 88.9 |
| Pteriomorphia | Pectinida | Pectinidae | *Mizuhopecten yessoensis* | 988Mb | 26415 | 1458 | 207 | 2123 | 7 | 8,3 | 98.6 |
| Pteriomorphia | Pectinida | Pectinidae | *Pecten maximus** | 918Mb | 26152 | 1615 | 370 | 1964 | 7,7 | 4,6 | 98.5 |
| Pteriomorphia | Mytiloida | Mytilidae | *Bathymodiolus platifrons* | 1.66Gb | 33584 | 1114 | 280 | 2045 | 5,2 | 2,3 | 51.4 |
| Pteriomorphia | Mytiloida | Mytilidae | *Modiolus philippinarum* | 2.6Gb | 36549 | 1060 | 323 | 2769 | 4,5 | 1,5 | 64.4 |
| Pteriomorphia | Mytiloida | Mytilidae | *Limnoperna fortunei* | 1.6Gb | 60717 | 916 | 298 | 3413 | 3,6 | 3,3 | 41.8 |
| Pteriomorphia | Mytiloida | Mytilidae | *Mytilus coruscus* | 1.9Gb | 64639 | 1281 | 253 | 2332 | 5 | 3,9 | 80.7 |
| Pteriomorphia | Mytiloida | Mytilidae | *Mytilus galloprovincialis* | 1.3Gb | 78735 | 1346 | 209 | 1878 | 6,5 | 8,3 | 80.3 |
| Euthyneura | Aplysiida | Aplysiidae | *Aplysia californica* | 927Mb | 19945 | 1708 | 321 | 2306 | 8,9 | 4,9 | 97.8 |

* in species column indicate chromosome-level assemblies.

**Table S3: Comparison between both *M. mercenaria* genome assemblies.**

|  | ***Mercenaria mercenaria*** | ***Mercenaria mercenaria YKG*** |
| --- | --- | --- |
| Total number of genes | 34728 | 34283 |
| Number of genes having a match against NR | 29892 (86) | 29761 (87) |
| Number of genes with no match againt NR (%) | 4836 (14) | 4522 (13) |
| Total number of BRH (%) | 23089 (66.5) | 23089 (67) |
| Number of genes unasigned in clusters | 2677 | 2753 |
| Number of specie-specific clusters | 480 | 335 |
| Number of genes in specie-specific clusters | 2952 | 1186 |
| Total number of genes not found in the other strain (%) | 5629 (16) | 3939 (11) |
| Total number of genes not found in the other strain with a match against NR (%) | 4095 (12) | 3273 (10) |
| Number of clusters with single copy genes | 16687 | 16687 |
| Number of clusters with both strains genes | 22287 | 22287 |
| Number of genes in clusters with both strains genes (%) | 29099 (84) | 30344 (89) |
| Number of orthologs defined by OrthoFinder | 24418 | 24418 |
| Number of orthologs having the same number of copies | 19369 | 19369 |
| Number of orthologs having more copies in strain | 1903 | 3146 |

Results generated from the functional annotation of both strains, BRH analysis and OrthoFinder analysis.

**Table S9: TNF-domain containing orthogroups.**

|  | Aplysiida | Mytiloida | | | | | Pectinida | | Ostreida | | | | Arcoida | Cardiida | Myida | Venerida | | | | |  |
| --- | --- | --- | --- | --- | --- | --- | --- | --- | --- | --- | --- | --- | --- | --- | --- | --- | --- | --- | --- | --- | --- |
| Orthogroup | *Aplysia californica* | *Mytilus coruscus* | *Mytilus galloprovincialis* | *Limnoperna fortunei* | *Modiolus philippinarum* | *Bathymodiolus platifons* | *Pecten maximus* | *Mizuhopecten yessoensis* | *Saccostrea glomerata* | *Ostrea edulis* | *Crassostrea gigas* | *Crassostrea virginica* | *Anadara broughtonii* | *Sinonovacula constricta* | *Dreissena rostriformis* | *Lutraria rhynchaena* | *Archivesica marissinica* | *Cyclina sinensis* | *Ruditapes philippinarum* | *Mercenaria mercenaria* (YKG) | Total |
| TNF Mollusca | |  |  |  |  |  |  |  |  |  |  |  |  |  |  |  |  |  |  |  |  |
| OG0000926 | 1 | 5 | 8 | 4 | 4 | 3 | 8 | 13 | 2 | 2 | 3 | 3 | 4 | 3 | 14 | 3 | 6 | 3 | 2 | 3 (3) | 94 |
| TNF Bivalvia | |  |  |  |  |  |  |  |  |  |  |  |  |  |  |  |  |  |  |  |  |
| OG0000639 | 0 | 3 | 9 | 2 | 5 | 2 | 0 | 0 | 1 | 2 | 1 | 1 | 3 | 5 | 11 | 14 | 4 | 6 | 9 | 24 (23) | 102 |
| OG0000960 | 0 | 1 | 2 | 1 | 0 | 0 | 0 | 0 | 0 | 1 | 1 | 1 | 3 | 0 | 0 | 16 | 1 | 6 | 11 | 22 (30) | 66 |
| OG0004827 | 0 | 0 | 0 | 1 | 1 | 1 | 0 | 0 | 0 | 0 | 0 | 0 | 0 | 8 | 2 | 2 | 1 | 15 | 1 | 2 (2) | 34 |
| OG0017573 | 0 | 0 | 0 | 0 | 1 | 0 | 0 | 0 | 0 | 0 | 0 | 0 | 0 | 0 | 0 | 0 | 0 | 2 | 2 | 2 (2) | 7 |
| TNF Heteroconchia | |  |  |  |  |  |  |  |  |  |  |  |  |  |  |  |  |  |  |  |  |
| OG0005640 | 0 | 0 | 0 | 0 | 0 | 0 | 0 | 0 | 0 | 0 | 0 | 0 | 0 | 0 | 0 | 1 | 1 | 3 | 3 | 12 (13) | 20 |
| OG0014042 | 0 | 0 | 0 | 0 | 0 | 0 | 0 | 0 | 0 | 0 | 0 | 0 | 0 | 0 | 0 | 1 | 2 | 6 | 2 | 2 (2) | 13 |
| OG0015779 | 0 | 0 | 0 | 0 | 0 | 0 | 0 | 0 | 0 | 0 | 0 | 0 | 0 | 0 | 0 | 2 | 2 | 2 | 1 | 2 (2) | 9 |
| OG0015781 | 0 | 0 | 0 | 0 | 0 | 0 | 0 | 0 | 0 | 0 | 0 | 0 | 0 | 2 | 0 | 1 | 1 | 1 | 2 | 2 (2) | 9 |
| TNF Specific to Mercenaria | | |  |  |  |  |  |  |  |  |  |  |  |  |  |  |  |  |  |  |  |
| OG0034212 | 0 | 0 | 0 | 0 | 0 | 0 | 0 | 0 | 0 | 0 | 0 | 0 | 0 | 0 | 0 | 0 | 0 | 0 | 0 | 2 (1) | 2 |
| OG0028962 | 0 | 0 | 0 | 0 | 0 | 0 | 0 | 0 | 0 | 0 | 0 | 0 | 0 | 0 | 0 | 0 | 0 | 0 | 0 | 1 (3) | 1 |
| OG0043344 | 0 | 0 | 0 | 0 | 0 | 0 | 0 | 0 | 0 | 0 | 0 | 0 | 0 | 0 | 0 | 0 | 0 | 0 | 0 | 1 (1) | 1 |
| OG0043346 | 0 | 0 | 0 | 0 | 0 | 0 | 0 | 0 | 0 | 0 | 0 | 0 | 0 | 0 | 0 | 0 | 0 | 0 | 0 | 1 (1) | 1 |

Number of genes containing TNF domain found in each related orthogroup, across 20 species from 8 families. Colors represent orthogroups used for the phylogenetic tree (Fig. 4).

**Table S10: Comparison between structural features of Steamer retrotransposons.**

| Steamer elements | Host species | copies with both LTRs | LTR start | LTR end | LTR size | PBS | putative PPT | Copies size | Consensus size | steamer copy of reference | | |  |
| --- | --- | --- | --- | --- | --- | --- | --- | --- | --- | --- | --- | --- | --- |
|  |  |  |  |  |  |  |  |  |  | contig, scaffold or chromosome | Start position - End position | ORF |  |
|  |  |  |  |  |  |  |  |  |  |  |  |  |  |
| Steamer | *Mya arenaria* |  | TGTAACA | ATAAACA | 177 | TGGTGTCAGAAG | AAAGGAAAAAGGA | 4968 | na | na | na | single ORF |  |
|  |  |  |  |  |  |  |  |  |  |  |  |  |  |
| SteMemer | *Mercenaria mercenaria* | 7 | TGTAACA | **T**TAAACA | 174 | TGGTGTCAGA**T**G | AAAAGAGAAAAGA | 4666-4678 | 4678 | chromosome_2 | 28255163 - 28259836 | single ORF |  |
| SteMemer | *Mercenaria mercenaria YKG* | 5 | TGTAACA | **T**TAAACA | 168-174 | TGGTGTCAGA**T**G | AAAAGAGAAAAGA | 4469-4678 | 4678 | Chr13 | 46412458 - 46417135 | single ORF |  |
| SteRuphi | *Ruditapes philippinarum* | 0 | - | A**G**AAACA | - | TGGTGTCAGAAG | - | - | na | QUSP01008422.1 | 414 - 4635 | na |  |
| SteCysin | *Cyclina sinensis* | 2 | TGTAACA | A**G**AAACA | 184 | TGGTGTCAGAAG | AAAAGAGAAAAGA | 4792-4802 | 4803 | JAAONU010000001.1 | 55805140 - 55809942 | single ORF |  |
| SteDrros | *Dreissena rostriformis* | 5 | TGTAACA | ATAAACA | 207-210 | TGGTGTCAGAAG | AAAAGAGAAAGGA | 4651-4742 | 4739 | scaffold1295 | 93751 - 98527 | corrupted |  |
|  |  |  |  |  |  |  |  |  |  |  |  |  |  |
| SteAnbro1 | *Anadara broughtonii* | 5 | TGTTACA | **CAT**AACA | 160-163 | TGGTGTCAGAAG | GAAAAAAGGGA | 4500-4700 | 4683 | Lachesis_group15 | 15699689 -15704399 | single ORF |  |
| SteAnbro2 | *Anadara broughtonii* | 5 | TGTAACA | **T**(A/T)**TT**ACA | 198-227 | TGGTGTCAGAAG | AAAGAAAAGGAGAT | 4226-4730 | 4739 | Lachesis_group4 | 40536963 - 40541655 | single ORF |  |
| SteAnbro3 | *Anadara broughtonii* | 5 | TGTAACA | **T**TA**T**ACA | 151-157 | TGGTGTCAGAA**T** | AAAAGGAAGGA | 4590-4633 | 4624 | Lachesis_group6 | 28502635 - 28507239 | 1 frameshift |  |
|  |  |  |  |  |  |  |  |  |  |  |  |  |  |
| SteCragi | *Crassostrea gigas* | 1 | TGTAACA | **TGAT**ACA | 204 | TGGTGTCAGAAG | AAAAAAAAAGGGAGA | 4890 | na | NC_047560.1 | 42383925 - 42388814 | 1 frameshift |  |
| SteCravi | *Crassostrea virginica* | 0 | TGTAACA | - | - | - | AAAAAAGGAGA | - | na | CM008249.1 | 4297195 - 4300438 | na |  |
| SteSaglo | *Saccostrea glomerata* | 4 | TGTAACA | **TG**A**T**ACA | 219 | TGGTGTCAGAAG | AAAAAAAAAGGGAGA | 4745-4925 | 4899 | PRKT01000085.1 | 1113877 - 1118774 | single ORF |  |
|  |  |  |  |  |  |  |  |  |  |  |  |  |  |
| SteBabla | *Bathymodiolus platifons* | 3 | TGTAACA | **T**TAAACA | 184-189 | TGGTGTCAGA**T**G | GAAAAAAGGAAGGA | 4195-4413 | 4543 | MJUT01005397.1 | 129884 - 134303 | corrupted |  |
| SteMophi | *Modiolus philippinarum* | 2 | TGTAACA | _**A**AAACA | 184-186 | TGGTGTCAG**GT**G | AAAAAAGAAAGGA | 4682-4692 | 4750 | MJUU01028173.1 | 30240 - 35566 | corrupted |  |
| SteLifor | *Limnoperna fortunei* | 1 | TGTAACA | **TA**TAACA | 198 | TGGTGTCAG**GT**G | AAAAAAGAAAGGA | 4784 | na | NFUK01001470.1 | 140079 - 144866 | corrupted |  |
|  |  |  |  |  |  |  |  |  |  |  |  |  |  |
| SteMycor1 | *Mytilus coruscus* | 2 | TGTAACA | ATAAACA | 186 | TGGTGTCAGA**T**G | GAAAAAGAAAGGA | 4672-4701 | na | CACVKT020010330.1 | 6113000 - 6118370 | 1 frameshift |  |
| SteMycor2 | *Mytilus coruscus* | 9 | TGTAACA | **T**TAAA**T**A | 187 | TGGTGTCAGA**T**G | AAAAGAGAAAGGA | 4707-4795 | 4790 | CACVKT020003686.1 | 174776 - 179537 | 2 frameshifts |  |
| SteMygal1 | *Mytilus galloprovincialis* | 1 | TGTAACA | ATAAACA | 185 | TGGTGTCAGA**T**G | GAAAAAAGAAAAGAAGA | 4698 | na | UYJE01005104.1 | 86319 - 91042 | 2 frameshifts |  |

Features from the reference *Steamer* element of *Mya arenaria* are from Metzger et al. 2018 (26).
